# Nonlinear Elasticity of Amorphous Silicon and Silica from Density Functional Theory

**DOI:** 10.1021/acs.jpcc.4c06550

**Published:** 2024-12-02

**Authors:** Umesh C. Roy, Angelo Bongiorno

**Affiliations:** † Department of Chemistry, College of Staten Island, Staten Island, New York 10314, United States; ‡ The Graduate Center of the City University of New York, New York, New York 10016, United States

## Abstract

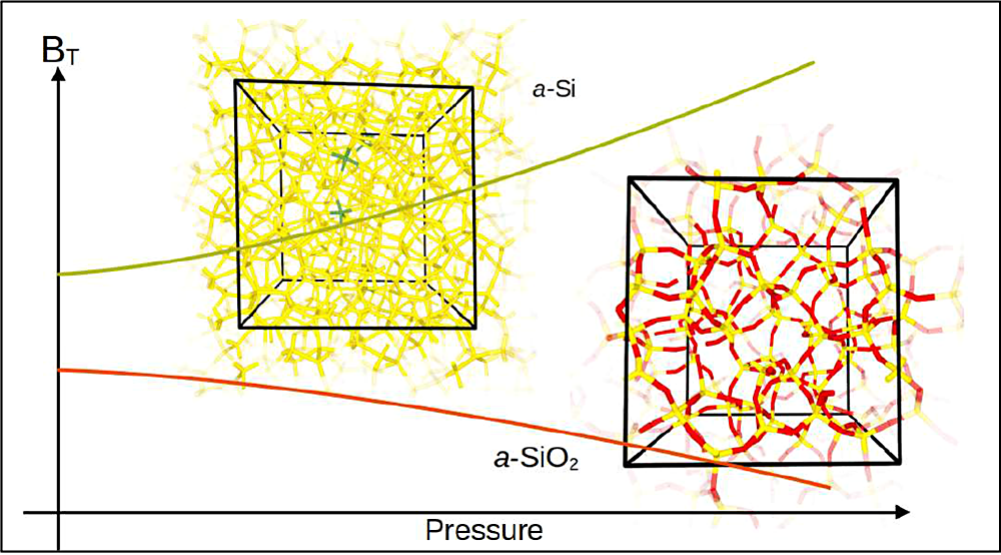

Density functional theory calculations and a finite deformation method are used to calculate second- and, most notably, third-order elastic constants of amorphous silicon and amorphous silicon dioxide, as represented by model structures generated via melt-quench force-field molecular dynamics simulations. Linear and nonlinear elastic constants are used to deduce macroscopic elastic moduli, such as the bulk and shear moduli, their pressure derivatives, and the elastic Grüneisen parameter. Our calculations show that the elastic properties of amorphous silicon reach the isotropic elastic limit within the nanometer length scale, attaining characteristics, both linear and nonlinear, comparable to those of crystalline silicon. In contrast, the nonlinear elastic properties of silica retain an anisotropic character over the nanometer length scales, yielding nonetheless the expected pressure-induced softening of the elastic moduli. This atypical elastic behavior is correlated to the occurrence of long-wavelength acoustic modes with negative Grüneisen parameters.

## Introduction

Amorphous solids are ubiquitous in technology,^1^ and understanding their mechanical properties is crucial to enable their applications, and to advance fundamental knowledge of the disordered state of materials.^2^ Prior to the onset of plasticity, a solid underloading undergoes finite and reversible elastic deformations. Although well characterized experimentally for selected metallic glasses^3−6^ and silica,^7−10^ the linear and nonlinear elastic regimes of amorphous materials remain overall poorly investigated, especially by atomistic computer simulations. Here, we use density functional theory (DFT) calculations to elucidate the nonlinear elastic properties of amorphous silicon (*a*-Si) and silica (*a*-SiO_2_), two disordered network-forming materials with technological applications in photovoltaics^11,12^ and microelectronics.^13^

Amorphous silicon is typically obtained in the form of a thin film, for example via chemical vapor deposition^14^ or sputtering.^15^ Measured values of Young’s and shear moduli show variations, depending on film preparation, hydrogen content, and the technique used to probe elastic properties.^16−21^ Notwithstanding, the reported values for these two elastic moduli are consistently 20–35% smaller than those of crystalline silicon, an effect commonly attributed to disorders. Nonlinear elastic properties are typically probed by using acoustoelastic techniques.^22^ These measurements require in general sizable sample dimensions, and to the best of our knowledge, they have not yet been conducted on thin films of *a*-Si. Although third-order elastic constants of *a*-Si remain unknown, ultrasonic measurements^23^ show that *a*-Si lacks the typical acoustic anomalies of other amorphous materials such as *a*-SiO_2_, thereby indicating that these two disordered network-forming materials exhibit different nonlinear elastic properties.

The elastic regime of *a*-SiO_2_ is well characterized, and both linear and nonlinear elastic coefficients have been determined experimentally.^10,24−31^ Interestingly, this amorphous solid exhibits an anomalous elastic behavior upon hydrostatic compression.^10,24−31^ In particular, experiments show that upon compression up to ∼2 GPa, both bulk and shear moduli of silica decrease,^10,24−31^ instead of increasing as typically occurs for the vast majority of materials.^32^ The origin of this uncommon elastic behavior remains to a large extent little understood.^10,25,27−29,33,34^ At pressures larger than ∼2 GPa, silica recovers a regular elastic behavior, which persists up to about 9 GPa, when anelastic and plastic events begin to occur.^26,28,31,35,36^

The linear elastic properties of both *a*-Si and *a*-SiO_2_ have been extensively studied by using atomistic computer simulations. Studies based on DFT calculations have employed model structures containing less than 200 atoms to describe these two amorphous solids and obtained elastic moduli at 0 K in overall good agreement with the experiment.^37−39^ Studies based on force fields^40−48^ and tight-binding^49^ methods have used, in general, larger model structures to calculate the elastic moduli of *a*-Si and *a*-SiO_2_. On several occasions, molecular dynamics (MD) simulations were also used to account for the role of temperature.^49^ Overall, the results of these computational studies are in satisfactory agreement with the experiment with systematic errors arising from the limited transferability of the interatomic potentials. Considerable work has been done to elucidate the nonlinear elastic properties of disordered soft-matter systems that can be described by Lennard–Jones or finite-range repulsive interatomic potentials.^50−56^ In contrast, to the best of our knowledge, similar computational studies of realistic amorphous materials such as *a*-Si and *a*-SiO_2_ have not yet been reported.

In this work, we use the environment-dependent interatomic potential (EDIP)^57−59^ and the force field developed by van Beest, Kramer, and van Santen (BKS)^60^ to generate via MD simulations and the conventional melt-quench approach model structures of *a*-Si and *a*-SiO_2_, respectively. The model structures of *a*-Si contain a small concentration of 5-fold coordinated Si atoms, whereas those of *a*-SiO_2_ are devoid of coordination defects. Then, we use a DFT approach and the finite deformation method developed by Cao et al.^61^ to calculate second- and third-order elastic constants of the two amorphous solids at zero pressure and zero temperature. Linear and nonlinear elastic constants are used to calculate macroscopic elastic moduli, i.e., bulk (*B*_*T*_), shear (*G*), and Young’s (*E*) moduli, and Poisson’s ratio (μ), the pressure derivative of these elastic coefficients, and the elastic Grüneisen parameter.^8,22^ Our results show that the two disordered network-forming materials exhibit distinct elastic attributes. The elastic properties of *a*-Si reach the isotropic elastic limit over length scales on the order of the nanometer, attaining characteristics comparable to those of crystalline silicon. At variance, while the linear elasticity of *a*-SiO_2_ converges rapidly in space, the nonlinear elastic properties retain an anisotropic character over the nanometer length scales. In agreement with the experiment, our calculations show that *a*-SiO_2_ exhibits the atypical elastic property of softening upon compression, a behavior that correlates with the occurrence of long-wavelength acoustic modes with negative Grüneisen parameters.

## Methods

### Amorphous Model Structures

**Figure 1:**
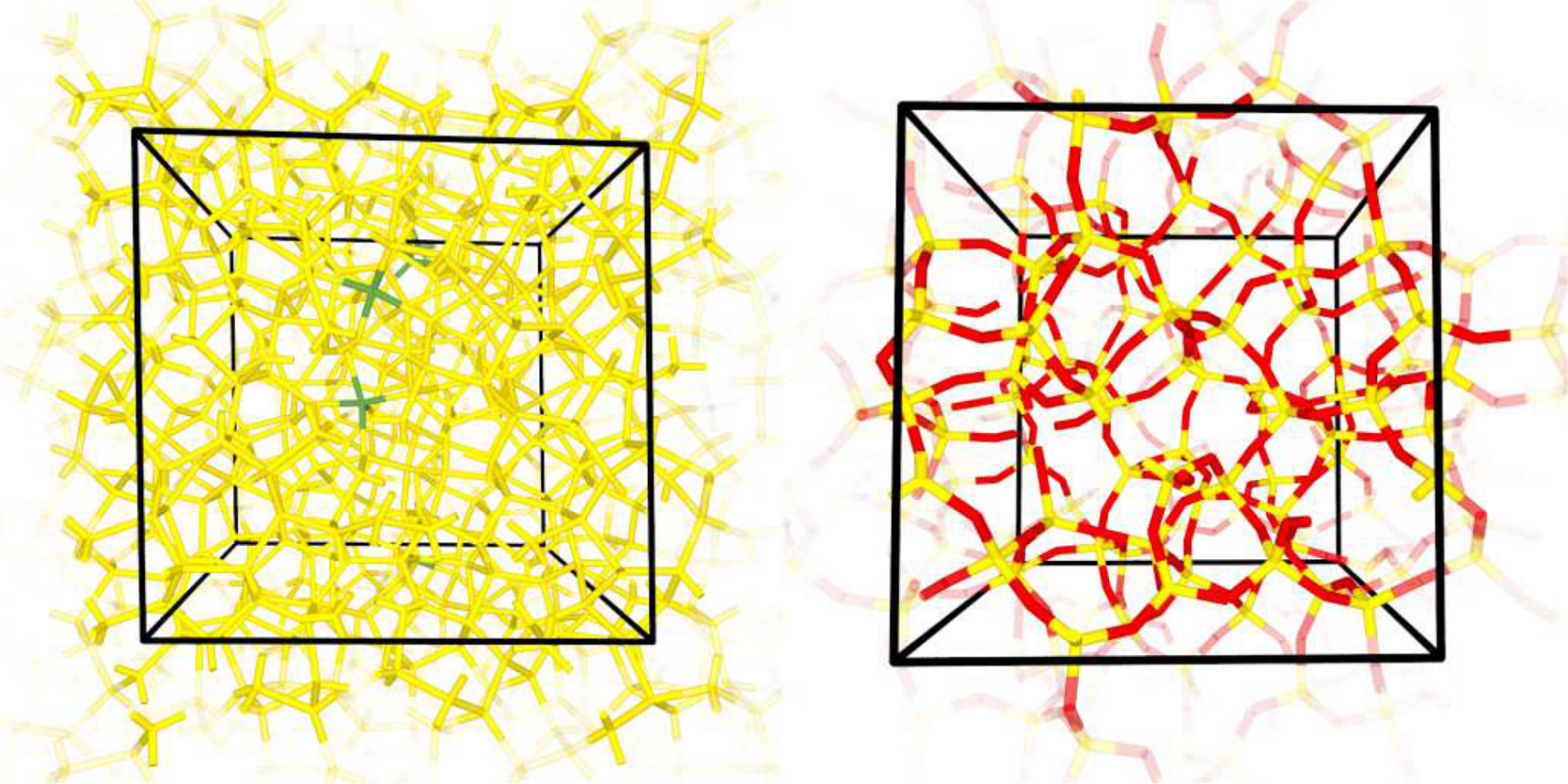
Images showing the disordered atomistic structure of models *a*-Si(1) (left) and *a*-SiO_2_(2) (right). The green sticks show coordination defects in *a*-Si(1).

To generate the model structures of *a*-Si and *a*-SiO_2_, we carry out canonical MD simulations using the EDIP^57−59^ and BKS^60^ interatomic potentials, respectively. MD simulations are carried out by using a proprietary code, implementing standard algorithms to describe a periodic atomistic system in the microcanonical or canonical ensembles, and the Ewald sums to treat electrostatic interactions.^62^ We generate 3 model structures of *a*-Si, each one containing 216 atoms, and with an initial cubic volume 1.8% smaller than in the crystalline phase.^20^ In the case of *a*-SiO_2_, we generate two model structures, containing 72 (*a*-SiO_2_(1)) and 144 (*a*-SiO_2_(2)) atoms, respectively. The mass density of these models is 2.2 g/cm^3^. In all cases, the melt-quench recipe consists of a thermalization run at 5,000 K for 100 ns, followed by quenching to 0 K at a rate of 100 K/ns. We then carry out a variable-cell DFT calculation^63,64^ to optimize both cell parameters and ionic positions and obtain model structures of *a*-Si and *a*-SiO_2_ at zero pressure and zero temperature (Figure 1). Cell parameters and atomic positions of the amorphous model structures are provided in the Supporting Information.

**Figure 2:**
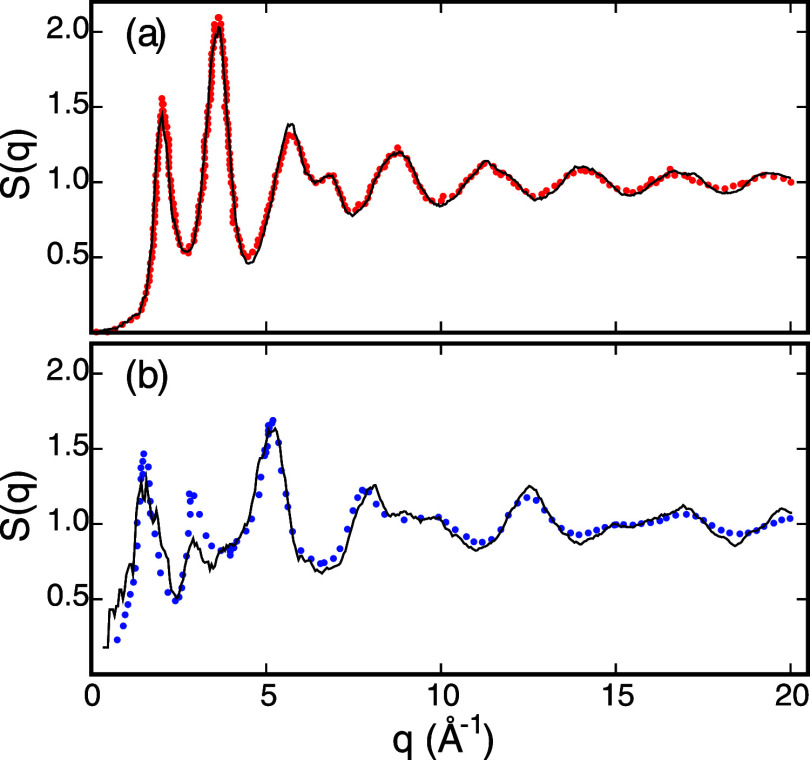
Static structure factors of (a) *a*-Si and (b) *a*-SiO_2_ versus the amplitude of the scattering wavevector. Black solid lines show spectra calculated by considering the model structures *a*-Si(1) and *a*-SiO_2_(2), whereas colored discs show neutron scattering data.^68,69^ For each model structure, the calculated spectrum is obtained by convoluting the Fourier transform of the static ionic configuration at 0 K with a rectangular function of width of 0.4 Å^–1^.

The three model structures of *a*-Si have the following structural features. The mass density is about 2.37 g/cm^3^. There are no 3-fold coordinated or undercoordinated Si atoms. The concentration of 5-fold coordinated Si atoms ranges from 2 to 4% (Figure 1), thereby yielding average coordination numbers of 4.03–4.05. Si–Si bond lengths are scattered around an average value of 2.34 Å with a variance of 0.05 Å, whereas bond angles are centered around 109° with a standard deviation of 11°. The two model structures of *a*-SiO_2_ preserve a mass density close to 2.2 g/cm^3^, they are devoid of coordination defects, and both have Si–O bond lengths, and Si–O–Si and O–Si–O bond angles distributed around 1.62 Å, 109.46°, and 143°, with variances of 0.01 Å, 3.50° and 13°, respectively. The model structure containing 72 atoms is devoid of three-fold rings and includes one 4-fold ring, whereas the larger model structure includes one 3-fold and several 4-fold rings. The aforementioned static structural properties are consistent with results from previous computational studies of *a*-Si and *a*-SiO_2_.^65−67^ Furthermore, our amorphous model structures exhibit static structure factors in agreement with neutron diffraction experiments (Figure 2).^68,69^ Overall, our analysis suggests that our amorphous model structures provide with a realistic description of *a*-Si and *a*-SiO_2_.

### Linear and Nonlinear Elastic Constants

**Figure 3:**
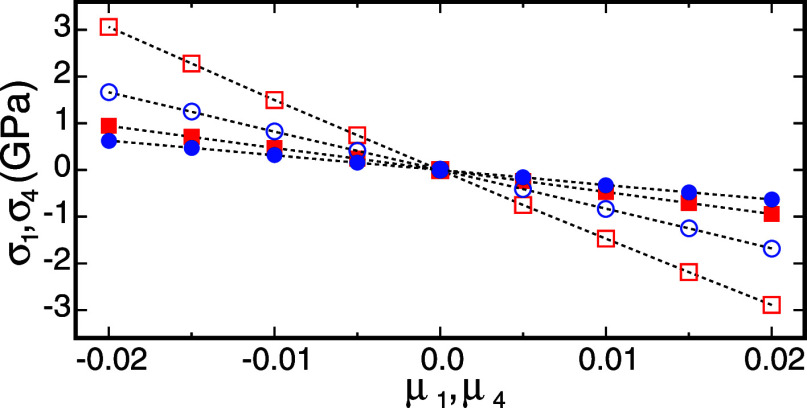
Selected components of the Cauchy stress tensor versus Lagrangian strain obtained from DFT by considering deformed configurations of *a*-Si(1) (red symbols) and *a*-SiO_2_(2) (blue symbols). Dashed lines are guides for the eye. Open symbols show the *xx* stress component versus the normal strain along the *x* axis, whereas filled symbols show the *yz* stress component versus the shear strain in the *yz* plane.

To calculate the second- and third-order elastic constants of *a*-Si and *a*-SiO_2_ (Figure 3), we use the method described in previous studies.^61,70^ This method involves three tasks. First, generating a list of deformed configurations by applying selected Lagrange strains to the reference state.^61,70^ Second, using a DFT approach^63,64^ to calculate the Cauchy stress tensor of each deformed configuration. Third, exploiting first- and second-order finite difference formulas to calculate second- and third-order elastic constants by numerical differentiation of the second Piola-Kirchhoff stress tensor.^61,70^ For completeness, to calculate, for example, *C*_*xxxxxx*_^(3)^, we use the following finite difference formula:
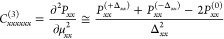
1where *P*_*xx*_^(±Δ_*xx*_)^ is the *xx* component of the second Piola-Kirchhoff stress tensor of the deformed configuration obtained by applying a normal uniaxial Lagrangian strain along the *x-*axis equal to ±Δ_*xx*_, and *P*_*xx*_^(0)^ is the stress tensor component of the undeformed reference state. Another example is the case of *C*_*xxyyzz*_^(3)^, which is obtained from the following second-order mixed finite difference formula:
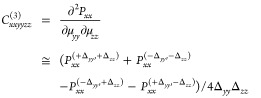
2where *P*_*xx*_^(±Δ_*yy*_, ± Δ_*zz*_)^ is the *xx* component of the second Piola–Kirchhoff stress tensor of the deformed configuration obtained by applying the combined Lagrangian strains along the *y* and *z* axes by ± Δ_*yy*_ and ± Δ_*zz*_, respectively. Thus, to recap, we use a first-order central finite difference formula,^61^ and either eq 1 or 2 to calculate the 21 independent second-order and 56 third-order elastic constants of our amorphous model structures, respectively. To accomplish this task, for each model structure we consider 85 deformed configurations, including the reference state.^61,70^

To optimize ionic positions and determine the Cauchy stress tensor of a deformed configuration, we use the *pw.x* code of the Quantum ESPRESSO software package.^63,64^ All DFT calculations are carried out by sampling the Brillouin zone at the Γ point and by employing the following convergence criteria: 10^–13^ Ry for self-consistency and 10^–3^ Ry/bohr for forces. To describe the electronic structure of *a*-Si, we use the norm-conserving pseudopotential *Si.pz-n-nc.UPF*,^71^ a local density approximation functional,^72^ and a plane-wave energy cutoff of 50 Ry. In the case of *a*-SiO_2_, we use the ultrasoft pseudopotentials *Si.pbesol-nl-rrkjus_psl.1.0.0.UPF* and *O.pbesol-nl-rrkjus_psl.1.0.0.UPF*,^71^ a generalized gradient approximation functional,^73^ and plane-energy energy cutoffs of 60 and 350 Ry for the wave functions and charge density, respectively. For completeness, we also use the EDIP^57−59^ and BKS^60^ interatomic potentials to calculate the linear and nonlinear elastic constants of *a*-Si and *a*-SiO_2_, respectively.

The nonaffine character of elastic deformations in amorphous solids poses both conceptual and technical challenges to atomistic modeling studies.^54−56,74−77^ Our method to calculate elastic constants is based on applying finite homogeneous strains to a reference supercell and using a DFT approach to accommodate the nonaffine displacement field and relieve the internal stress caused by the deformation. To ensure that the finite strains applied to our model structures probe the elastic regime of the amorphous configuration and do not trigger plastic events, we carry out our preemptive DFT calculations to determine the optimal strain parameter to calculate elastic constants via our finite-deformation method. Figure 2 shows selected stress versus strain curves obtained by applying normal and shear Lagrangian strains between −0.02 and +0.02 to our model structures of *a*-Si and *a*-SiO_2_. These curves demonstrate that strains up to ±0.02 are sufficiently small to induce reversible elastic deformations and preserve the structural integrity of our amorphous model structures. Based on this result, we use a strain parameter of 0.015 to generate the list of deformed configurations necessary to calculate the full set of second- and third-order elastic constants (eqs 1 and 2). The full list of linear and nonlinear elastic constants for all model structures obtained from our DFT calculations is provided in the Supporting Information.

The model structures used in this study have a moderate size, and the reference states at zero pressure yielded by the variable-cell optimization calculations have triclinic geometry. As a consequence, all the 21 and 56 independent second- and third-order elastic constants of our model structures are nonzero, and their values do not reflect in full the isotropic nature of an amorphous solid. Thus, to reduce the impact of size effects and deduce isotropic elastic constants, we carry out the following averaging operation:
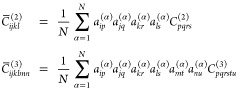
3where *C*_*pqrs*_^(2)^ and *C*_*pqrstu*_^(3)^ are the second- and third-order elastic constants obtained from numerical differentiation of the second Piola-Kirchhoff stress tensor, *C̅*_*ijkl*_^(2)^ and *C̅*_*ijklmn*_^(3)^ are the symmetrized elastic constants, and *a*_*ij*_^(α)^ is a 3 × 3 rotation matrix. The averaging operation in eq 3 is carried out by considering a large number (*N* ∼ 2000) of random proper rotations.

## Discussion

The symmetrized values of the second- and third-order elastic constants for the three model structures of *a*-Si are reported in Table 1. For convenience, henceforth we use the Voigt notation to refer to distinct elastic constants (see the caption of Table 1). For completeness, Table 1 reports also experimental data, present and previous computational estimates of the independent second- and third-order elastic constants of crystalline silicon,^70,78,79^ as well as results obtained by using the EDIP potentials. Table 1 shows that the values for *C̅*_11_^(2)^, *C̅*_12_^(2)^, and *C̅*_44_^(2)^ obtained from DFT are, on average, smaller than those obtained for crystalline silicon by 5, 10, and 37%, respectively. In overall accord with previous computational studies, these results show that in the case of *a*-Si, since its mass density is very similar to that of the crystalline phase, the softening of the second-order elastic constants is attributed solely to disorder. As for the third-order elastic constants, our DFT results show that similar to crystalline silicon, *C̅*_111_^(3)^ retains a large negative value, whereas *C̅*_144_^(3)^ and *C̅*_456_^(3)^ remain small and negligible. In contrast, compared to crystalline silicon, *C̅*_112_^(3)^ and *C̅*_155_^(3)^ soften by about 50 and 67%, respectively, whereas *C̅*_123_^(3)^ hardens its negative value by about 120 GPa. Table 1 shows also that, as compared to both the experiment and DFT, the EDIP potentials significantly underestimate and overestimate the *C*_112_^(3)^ and *C*_123_^(3)^ of crystalline silicon, respectively. Such faultiness in the case of the ordered phase is passed on to disordered Si, as shown by the discrepancies between the third-order elastic constants obtained from EDIP and DFT. We remark that, in all cases, the symmetrization operation in eq 3 yields elastic constants satisfying the relationships valid for isotropic materials, i.e.,
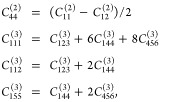
4thus reducing the number of independent second- and third-order elastic constants to 2 and 3, respectively.

**Table 1:** Symmetrized Second- and Third-Order Elastic Constants (in GPa) of *a*-Si Were Obtained from DFT and EDIP Calculations^a^

	11	12	44	111	112	123	144	155	456
crystalline silicon									
exp. (78)	166	64	80	–795	–445	–75	15	–310	–86
ref (70)	153	57	75	–751	–423	–78	16	–294	–59
ref (79)	152	59	78	–653	–456	–96	23	–304	–7
DFT	162	64	77	–777	–461	–83	31	–299	–51
EDIP	172	65	72	–940	–29	432	6	–290	3
amorphous silicon: DFT									
*a*-Si(1)	147	52	48	–688	–272	–206	–33	–104	–35
*a*-Si(2)	142	51	46	–594	–243	–198	–22	–88	–33
*a*-Si(3)	148	50	49	–754	–264	–168	–45	–123	–37
amorphous silicon: EDIP									
*a*-Si(1)	133	83	25	–186	61	146	–43	–61	–10
*a*-Si(2)	136	82	27	–167	46	105	–30	–53	–12
*a*-Si(3)	136	79	28	–98	128	216	–43	–57	–6

a Experimental data, present and previous computational results for crystalline silicon are also reported. Voigt indices (ranging from 1 through 6, each one indicating a pair of Cartesian indices, as follows: 1 → *xx*, 2 → *yy*, 3 → *zz*, 4 → *yz*, 5 → *zx*, and 6 → *xy*) in the first row, αβ and αβγ, are used to refer to second- and third-order elastic constants, *C̅*_αβ_^(2)^ and *C̅*_αβγ_^(3)^, respectively.

Table 2 reports the computed symmetrized elastic constants for the two model structures of *a*-SiO_2_, along with available experimental data.^8,80^Table 2 shows that second-order elastic constants obtained from DFT by using the 72-atom model structure (*a*-SiO_2_(1)) deviate by up to 20% from experimental data, whereas a much better agreement is obtained by using the model structure containing 144 atoms. In both cases, the symmetrized second-order elastic constants satisfy the isotropy conditions in eq 4. These results suggest that the linear elastic properties of *a*-SiO_2_ converge rapidly in space, attaining an isotropic limit over length scales on the order of the nanometer. As expected, similar results are also obtained by using the BKS potentials, known to reproduce well the elastic properties of both the crystalline and amorphous phases of SiO_2_.^60^

**Table 2:** Symmetrized Second- and Third-Order Elastic Constants (in GPa) of *a*-SiO_2_ Obtained from DFT and (Last Two Rows) BKS Interatomic Potentials^a^

	11	12	44	111	112	123	144	155	456
exp. (8)	78	31	16	526	239	54	93	72	–11
exp. (80)				371	270	–77	75	66	–66
*a*-SiO_2_(1)	96	20	38	110	155	52	50	–10	–32
*a*-SiO_2_(2)	79	21	29	149	28	–53	37	32	–53
*a*-SiO_2_(1)	110	28	41	120	150	8	150	70	–39
*a*-SiO_2_(2)	77	22	28	359	217	103	55	36	–11

a Experimental data are reported for comparison. Voigt indices are used to refer to second- and third-order elastic constants.

An isotropic material has three independent third-order elastic constants, typically *C*_123_^(3)^, *C*_144_^(3)^, and *C*_456_^(3)^. The remaining three elastic constants satisfy the relationships in eq 4.^8,81^Table 2 shows that the symmetrization operation in eq 3 yields third-order elastic constants that do not fully satisfy the isotropy condition in eq 4. For example, the value of *C̅*_111_^(3)^ obtained for *a*-SiO_2_(2) from the second relationship in eq 4 using DFT results is 117 GPa, whereas the value obtained from the symmetrization operation is 149 GPa. These deviations from isotropicity suggest that the size of our model structures for *a*-SiO_2_ is inadequate to reproduce in full and quantitatively the nonlinear elastic properties of *a*-SiO_2_.

Both experimental and computed third-order elastic constants in Table 2 are small in value. Experimental data show some scattering, whereas our computed values suffer from limited statistics and moderate size of the amorphous model structures. Nonetheless, the comparison in Table 2 leads to an interesting result. Third-order elastic constants can be used to calculate the pressure derivatives of bulk and shear moduli (see ref (32)), and for both model structures, we obtain negative values for these nonlinear elastic coefficients (Table 3). Experimental data for *B*_*T*_*′* and *G′* are scattered around −6 and −3, respectively,^10^ and our estimates are smaller in value (Table 3). Nonetheless, considering that the pressure derivative of *B*_*T*_ and *G* is mainly controlled by *C̅*_111_^(3)^ and *C̅*_112_^(3)^, the accord with the experiment demonstrates that both sign and value of the dominant third-order elastic constants are qualitatively well reproduced by our calculations. We remark that this result is not an artifact of the symmetrization operation in eq 4. In fact, similar values for *B*_*T*_*′* and *G′* are also obtained by using the unprocessed third-order elastic constants of the two model structures of *a*-SiO_2_. We remark that a similar conclusion can be drawn using the results obtained using the BKS potentials (Table 4).

**Table 3:** Bulk, Young’s, and Shear Moduli (in GPa), and Poisson’s Ratio, and Their Pressure Derivatives of *a*-Si and *a*-SiO_2_ Derived from the Second- and Third-Order Elastic Constants in Tables 1 and 2, As Described in Ref (32)

	*B*_*T*_	*E*	*G*	ν	*B*_*T*_*′*	*E′*	*G′*	*ν′*
*a*-Si(1)	83	120	48	0.26	3.64	0.36	–0.23	0.01
*a*-Si(2)	81	116	46	0.26	3.34	–0.04	–0.37	0.01
*a*-Si(3)	83	123	49	0.25	3.59	0.85	–0.02	0.01
*a*-SiO_2_(1)	46	89	38	0.18	–2.80	–3.92	–1.50	0.00
*a*-SiO_2_(2)	40	67	29	0.21	–0.58	–4.25	–2.07	0.01

**Table 4:** Elastic Moduli (in GPa) and Their Pressure Derivative of *a*-Si and *a*-SiO_2_ Derived From Elastic Constants Computed by Using EDIP and BKS Potentials, Respectively

	*B*_*T*_	*E*	*G*	ν	*B*_*T*_*′*	*E′*	*G′*	*ν′*
*a*-SiO_2_(1)	55	98	41	0.20	–2.08	–3.79	–1.58	0.00
*a*-SiO_2_(2)	40	67	27	0.22	–5.16	–6.17	–2.29	–0.01
*a*-Si(1)	99	69	25	0.38	–0.53	–1.38	–0.53	0.00
*a*-Si(2)	100	73	27	0.38	–0.36	–1.63	–0.64	0.00
*a*-Si(3)	98	78	28	0.37	–1.24	–1.50	–0.56	0.00

Tables 3 and 4 report values of bulk, Young’s, and shear moduli, and Poisson’s ratio of *a*-Si and *a*-SiO_2_, derived from the symmetrized second-order elastic tensors of our amorphous model structures. Overall, the DFT results are in good agreement with the experiment,^8,20,21^ and the values obtained by using the force fields show the expected discrepancies. Tables 3 and 4 report also the pressure derivatives of the aforementioned elastic moduli. These nonlinear elastic coefficients can be deduced, numerically, by employing elastic constitutive equations based on both second- and third-order elastic constants.^32^ While experimental data for these nonlinear elastic coefficients are absent for *a*-Si, as discussed above our results for *a*-SiO_2_ are in qualitative agreement with the experiment, reporting negative values for the pressure derivatives of both the bulk and shear moduli.^10,24−31^ Interestingly, contrary to the case of *a*-SiO_2_, our DFT calculations yield positive values of *B*_*T*_*′* for *a*-Si, suggesting that these two disordered network-forming solids exhibit different nonlinear elastic responses, in agreement with ultrasonic absorption and sound velocity measurements at low temperatures.^23^ We also remark that the value of *B*_*T*_*′* obtained for crystalline silicon by using EDIP potentials is 0.28, significantly smaller than the experimental and DFT result of about 4.2.^32^ This discrepancy further underlines the limitations of EDIP to describe nonlinear elastic properties of both ordered and disordered phases of Si.

It is interesting to make a connection between nonlinear elastic behaviors under pressure and anharmonic dynamical properties. In particular, we can use second- and third-order elastic constants to estimate, within a continuum approximation, the (elastic) Gruneisen parameters of *a*-Si and *a*-SiO_2_. To this end, we rely on the formulation developed by Brugger^8,22^ to calculate the generalized mode Grüneisen parameters of long-wavelength acoustic modes of frequency ω_*p*_(*q⃗*) = *v*_*p̂*_(*q⃗*) *q*, where *q⃗* is the propagation wave vector, and *v*_*p̂*_ is the sound velocity of the transverse (*p* = 1, 2) and longitudinal (*p* = 3) waves with polarization unit vector *p̂*. In terms of the zero-temperature symmetrized second- and third-order elastic constants computed in this study, the generalized Grüneisen parameters can be written as follows:
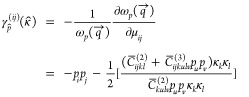
5where κ̂ = *q⃗*/*q*, and *p*_*i*_ and κ_*i*_ are coordinates of the polarization and propagation (unit) vectors *p̂* and κ̂, respectively. Here, we use this definition to calculate the average (elastic) Grüneisen parameters:
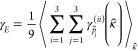
6where the averaging is carried out over the full solid angle of propagation directions of the long-wavelength sound waves. By combining eqs 5 and 6, and using the elastic constants computed from DFT in Tables 1 and 2, we obtain values of γ_*E*_ equal to 0.02 and −1.28 for *a*-Si and *a*-SiO_2_, respectively. These values suggest that contrary to *a*-Si, *a*-SiO_2_ is rich in low-energy acoustic modes exhibiting negative mode Grüneisen parameters, i.e., modes that become softer upon compression. This result is consistent and correlates well with the elastic responses to hydrostatic pressure exhibited by *a*-Si and *a*-SiO_2_. We remark that similar results are obtained for *a*-SiO_2_ by employing elastic constants calculated using the BKS potentials. In particular, we obtain values of γ_*E*_ equal to −1.23 and −1.99 for *a*-SiO_2_(1) and *a*-SiO_2_(2), respectively.

## Conclusions

We used force fields and a melt-quench MD approach to generate model structures of *a*-Si and *a*-SiO_2_, respectively, poor in and devoid of coordination defects. Then, we used a finite-deformation approach to calculate the second- and, most notably, third-order elastic constants of these amorphous solids, and hence to deduce macroscopic elastic moduli, their pressure derivatives, and the elastic Grüneisen parameter. Our DFT results show that *a*-Si and *a*-SiO_2_ exhibit distinct elastic attributes. Linear and nonlinear elastic properties of *a*-Si attain the isotropic elastic limit over length scales smaller than the nanometer, overall preserving characteristics comparable to those of crystalline silicon, such as a positive pressure derivative of the bulk modulus. In contrast, *a*-SiO_2_ exhibits nonlinear elastic properties converging to the isotropic limit over length scales larger than the nanometer. Furthermore, contrary to the case of α-quartz,^32^ in agreement with the experiment our results show that *a*-SiO_2_ exhibits the atypical property of softening upon compression, a behavior that correlates with the occurrence of long-wavelength acoustic modes with negative Grüneisen parameters.

## Supporting Information Available

Cell parameters and atomic positions of the amorphous model structures generated in this study; corresponding lists of second- and third-order elastic constants; and “README”file included to provide details about files, their content, and formatting (PDF)
