# Supplementary material for: Nonlinear Elasticity of Amorphous Silicon and Silica from Density Functional Theory
Source: J Phys Chem C Nanomater Interfaces. 2024 Dec 2;128(49):21220–7. doi: 10.1021/acs.jpcc.4c06550 (PMC11648081; doi:10.1021/acs.jpcc.4c06550)
Supplement: Supplementary file 1 — jp4c06550_si_001.pdf [file jp4c06550_si_001.pdf]

Supporting Information for

"Nonlinear Elasticity of Amorphous Silicon and Silica from Density Functional Theory"

Umesh C. Roy and Angelo Bongiorno

Department of Chemistry, College of Staten Island,
Staten Island, NY 10314

The Graduate Center of the City University of New York,
New York, NY 10016

```
--------------------------------------------------
 README
--------------------------------------------------
 Cell parameters (in bohr) and atomic positions
 (fractional coordinates) of the model structures
 of amorphous silicon and amorphous silicon dioxide,
 followed by the corresponding list of second- and
 third-order elastic constants obtained in this
 study. File naming follows the notation used in
 the main paper. Useful details about data formatting:

 CELL_PARAMETERS (bohr)
  a1_x a1_y a1_z
  a2_x a2_y a2_z
  a3_x a3_y a3_z
 ATOMIC_POSITIONS (crystal)
 Symbol   x y z
   .      . . .
   .      . . .


 SOECs (Second-Order Elastic Constants)

        value  voigt indices

  e.g.  149.00  11
          .     .

 TOECs (Third-Order Elastic Constants)

        value  voigt indices

  e.g. -703.62  111
          .     .

--------------------------------------------------
END README
--------------------------------------------------
```

```
--------------------------------------------------
 a-Si(1) - model structures
```

```
--------------------------------------------------
CELL_PARAMETERS (bohr)
  30.601676240   0.289074158   0.457182853
   0.289439143  30.991456536   0.213289941
   0.455502004   0.213386719  30.173744180
ATOMIC_POSITIONS (crystal)
Si               0.1763703079        0.2747455084        0.2034124704
Si               0.0356049371        0.3308176115        0.0364402663
Si               0.2812033685        0.0964132562        0.0576868248
Si               0.0211222339        0.1507394835        0.1788573123
Si               0.2067966448        0.2185868599        0.0693806625
Si               0.4798317263        0.4374144030        0.0393486135
Si               0.0385810155        0.2927372752        0.1756190307
Si               0.4099855569        0.1166957180        0.1213245124
Si               0.4671178557        0.2423232757        0.1425088710
Si               0.4023092495        0.4802817932        0.1607350365
Si               0.4293883110        0.3069185384        0.0183388313
Si               0.1340791902        0.0733361851        0.2324458486
Si               0.2402969883        0.4567677775        0.0103256844
Si               0.2868435301        0.3209584570        0.0060878838
Si               0.4896064726        0.0796101392        0.0047332944
Si               0.4551823357        0.2987803555        0.2783318020
Si               0.1125649141        0.4173497642        0.3700686534
Si               0.2005593315        0.1731855441        0.3085519218
Si               0.3542981639        0.4599151208        0.4111904686
Si               0.2104059808        0.4634431754        0.4665710161
Si               0.1323809125        0.2028442954        0.4396945333
Si               0.3318667905        0.1172383869        0.3452313696
Si               0.4219722752        0.2133714642        0.3933140046
Si               0.0356950843        0.3075689329        0.4228939109
Si               0.3558427055        0.4016894515        0.2772965438
Si               0.3074239393        0.0324292711        0.4624073196
Si               0.2147432690        0.3945627412        0.2630817768
Si               0.0273713992        0.1136462171        0.4860422972
Si               0.0047794210        0.4913772502        0.3029257866
Si               0.4442236877        0.3888779983        0.4979871971
Si               0.4167701065        0.0448106391        0.2473170915
Si               0.1794885057        0.3827041191        0.5865456218
Si               0.2106071551        0.0359006240        0.5762223950
Si               0.0413915760        0.3860284563        0.6379158148
Si               0.3458906785        0.1320728604        0.7119501961
Si               0.2487649050        0.4527676445        0.6904424074
Si               0.2156825268        0.1536050071        0.6510426718
Si               0.4269329037        0.2180143557        0.6328199271
Si               0.2206696063        0.2530614979        0.5453607360
Si               0.4253093584        0.0248841361        0.5358101260
Si               0.4567911259        0.4794548038        0.6065040034
Si               0.4551759273        0.4150109814        0.7407723300
Si               0.3636940830        0.2729205916        0.5159552312
Si               0.0669700164        0.4584110704        0.8499611705
Si               0.3491082926        0.1684978797        0.8509772506
Si               0.2721063338        0.2883879976        0.8667452090
Si               0.1383856424        0.2458804791        0.8445334810
Si               0.3238032714        0.4041211159        0.7993574059
Si               0.2944267307        0.0511036555        0.9148319077
Si               0.0433011419        0.3382847643        0.7817128466
Si               0.4686235590        0.1998079020        0.9233459957
Si               0.1283107777        0.1300951479        0.7663027393
Si               0.1008587026        0.4466897479        0.9896783107
Si               0.0903099463        0.2120440601        0.9827121365
```

Si	0.1933260262	0.0065169470	0.8097097244
Si	0.1618456283	0.8837618666	0.0857707251
Si	0.3824615602	0.7540206654	0.0316088271
Si	0.2609329597	0.6797008946	0.0549257280
Si	0.0617574235	0.7050496960	0.1982737747
Si	0.1889351704	0.7588045985	0.1534973978
Si	0.0704938001	0.5653217691	0.1967386147
Si	0.2853466450	0.5471063463	0.1096846863
Si	0.4252884885	0.8868332769	0.0730257154
Si	0.2953569328	0.7347153879	0.2424297562
Si	0.3480693793	0.9366823777	0.1937051741
Si	0.4201524146	0.6958294419	0.1704153905
Si	0.0289932350	0.5482968606	0.0601440411
Si	0.2053758020	0.5348901692	0.2308937649
Si	0.0198810041	0.8929469935	0.0968433406
Si	0.4647517737	0.5740671329	0.2422599226
Si	0.2248891848	0.9959398949	0.1446474572
Si	0.2802786521	0.9011287616	0.4247999526
Si	0.3451241294	0.8456100021	0.3058232580
Si	0.4765557805	0.7922004817	0.3318772298
Si	0.0950066882	0.7824885078	0.4311631306
Si	0.1361418342	0.9164410861	0.4222974632
Si	0.3928009707	0.5892772560	0.3689285968
Si	0.0640874065	0.9811790979	0.3156422583
Si	0.2607009432	0.6291979487	0.3292451612
Si	0.2118150079	0.6977362746	0.4490034765
Si	0.4800478890	0.6846212397	0.4297944874
Si	0.0152995249	0.7741347936	0.3135342063
Si	0.2992301074	0.7892158449	0.5173300118
Si	0.4286652476	0.7345819626	0.5577741215
Si	0.4575758543	0.9004818610	0.7244451430
Si	0.3268458013	0.5487914091	0.6147043475
Si	0.2443281209	0.9437787656	0.6828892396
Si	0.3019918788	0.7169083171	0.7405507904
Si	0.1051548023	0.6004912729	0.6446085730
Si	0.2307133149	0.8035573787	0.6484247810
Si	0.2079898227	0.5856262673	0.5384332332
Si	0.0179870497	0.7876168793	0.5558426401
Si	0.1038691094	0.7396807153	0.6571724021
Si	0.4086959729	0.6457034999	0.6741997896
Si	0.3743189595	0.9983672527	0.6700616245
Si	0.0852370193	0.9932950777	0.5379452840
Si	0.0105815257	0.9098134196	0.6307750935
Si	0.1811268245	0.8470401881	0.9446204020
Si	0.4382751140	0.5368708281	0.9459791122
Si	0.2350116780	0.6249559292	0.8320593479
Si	0.4181653915	0.9722869761	0.9403070692
Si	0.3270686734	0.8506827182	0.9270041503
Si	0.0900519048	0.9130472582	0.8497532255
Si	0.3884029264	0.8032287440	0.8049041293
Si	0.1778941198	0.7015348948	0.9418404813
Si	0.4831413367	0.6685473207	0.9709347576
Si	0.3086181595	0.5143591521	0.8930131092
Si	0.0452478746	0.6719740513	0.9871888757
Si	0.1533808981	0.5349870999	0.7615215282
Si	0.0368229809	0.8264620158	0.7494029536
Si	0.8090147096	0.2949005418	0.0612019000
Si	0.8144691335	0.3621378547	0.1877072821
Si	0.8039882642	0.1156568466	0.1940801250
Si	0.6246658804	0.4389364645	0.0408700913

Si	0.8408453690	0.1591344727	0.0583375981
Si	0.6049642244	0.2067212302	0.1088564934
Si	0.6226690127	0.0778951889	0.0520890823
Si	0.9834352307	0.1265967718	0.0430007862
Si	0.6725065101	0.3043241685	0.0230266680
Si	0.6823021690	0.1853663423	0.2276100547
Si	0.9429282282	0.3733402388	0.2483041246
Si	0.7808435015	0.3007414730	0.4054143524
Si	0.7116224748	0.3094875584	0.2793815910
Si	0.9596626531	0.0576027188	0.3781995075
Si	0.5820719501	0.3357590340	0.4678798061
Si	0.6301196027	0.1145764595	0.3443278396
Si	0.7328006849	0.0670819592	0.4352235818
Si	0.9161286511	0.2773594276	0.3498189991
Si	0.5785469652	0.3639260657	0.3166530656
Si	0.5556530508	0.1934978149	0.4415907602
Si	0.9135572663	0.1509553241	0.2794465082
Si	0.5536964571	0.0108027476	0.2874740485
Si	0.7131509014	0.4202137185	0.4632037948
Si	0.5859854972	0.5011710476	0.2795201539
Si	0.6205541536	0.0815351827	0.6431127913
Si	0.6224666390	0.3199029198	0.6161688011
Si	0.9539462361	0.3242108245	0.5445411824
Si	0.9199110134	0.1864647345	0.5525524271
Si	0.6681719089	0.4376062307	0.6829910944
Si	0.6997191777	0.1972735185	0.6202397318
Si	0.9006505858	0.1123502191	0.6743907278
Si	0.8327877234	0.4101345908	0.5477980739
Si	0.8078361315	0.4150760032	0.6935423306
Si	0.5164795491	0.2896622115	0.7113532390
Si	0.7837889744	0.1740895979	0.7332344537
Si	0.5128189198	0.1285278276	0.5645568003
Si	0.7831661933	0.1857254384	0.4985963327
Si	0.5734427747	0.2085623338	0.8204447557
Si	0.7481702008	0.0689355063	0.8303875150
Si	0.6090758028	0.0688092503	0.7899790806
Si	0.6379922702	0.4274550281	0.8270287096
Si	0.8549780221	0.4529914347	0.9403484495
Si	0.8043609853	0.3000201315	0.7907460803
Si	0.6744181491	0.2954823039	0.8725607359
Si	0.9131083204	0.3280415453	0.9667814662
Si	0.9346030664	0.2606186773	0.8395764465
Si	0.7510829104	0.0812518722	0.9781728425
Si	0.7134836712	0.4835194483	0.9296625705
Si	0.9841790000	0.1248231579	0.7925943453
Si	0.5405403051	0.7577861933	0.2020298359
Si	0.7690619594	0.6985191906	0.2041800372
Si	0.7713818143	0.9786935233	0.1795919867
Si	0.8855091058	0.5294613670	0.0556359225
Si	0.5648474925	0.8700640587	0.1125601951
Si	0.8435512361	0.7451434641	0.0847124521
Si	0.6405246256	0.6616294020	0.1555588850
Si	0.6573542922	0.5185287254	0.1535081164
Si	0.9865212455	0.7574686451	0.0872512350
Si	0.8144674544	0.9672770628	0.0390528964
Si	0.6182449548	0.6837588242	0.0152392120
Si	0.6279034516	0.9836344107	0.1639033014
Si	0.9840284550	0.8959947374	0.2406295442
Si	0.9538279465	0.9917570618	0.0139151171
Si	0.7975695882	0.4980552402	0.1672573572

```
Si              0.6744899028      0.8035672400      0.4042991843
Si              0.8996993781      0.7204570358      0.3779543020
Si              0.8444190570      0.9093353011      0.2814093881
Si              0.5628296679      0.8915793924      0.3710842197
Si              0.6255081011      0.6681639676      0.4327290385
Si              0.8009876081      0.5774270226      0.2838857366
Si              0.7873870297      0.7846158355      0.3141219463
Si              0.6854965958      0.5462417820      0.3717242387
Si              0.9098444250      0.5760564077      0.3759152618
Si              0.8461773141      0.9784609646      0.4095377345
Si              0.6657317811      0.6552812037      0.5700238937
Si              0.8267856448      0.6541061738      0.7175438000
Si              0.8099854441      0.6508271036      0.5738690281
Si              0.6497656332      0.7830163433      0.6357783428
Si              0.8898636317      0.5264419196      0.7223134408
Si              0.5884209876      0.5475478976      0.6303391648
Si              0.7091612694      0.8634891790      0.5323481069
Si              0.5172420031      0.8338510616      0.6080255296
Si              0.8439260931      0.8958156185      0.5306221004
Si              0.8816774859      0.9714179192      0.6517689419
Si              0.8868921799      0.7590271793      0.5164481848
Si              0.5291225335      0.9344636637      0.5037443438
Si              0.8869858751      0.5394409789      0.5172940960
Si              0.5455575056      0.6206237105      0.7494156003
Si              0.9910755754      0.5227639743      0.6169463886
Si              0.6528109408      0.9935147823      0.5351758325
Si              0.7825074481      0.8399916792      0.9920717631
Si              0.8443196371      0.9611306266      0.7966461019
Si              0.5233738293      0.9587546118      0.8336210628
Si              0.5898016951      0.8387512292      0.8667741449
Si              0.9139332432      0.7523158802      0.7839020320
Si              0.6950490554      0.6531484600      0.7851558298
Si              0.7145383325      0.6230645363      0.9250742332
Si              0.9462293380      0.6601319657      0.8893526602
Si              0.8095573431      0.8331643665      0.8433671270
Si              0.6921371910      0.7934161035      0.7754750515
Si              0.9421932231      0.5253082163      0.8516424085
Si              0.8393088439      0.6456533200      0.9868851481
Si              0.5044015251      0.7296561651      0.8405597974
Si              0.5175291137      0.5036153246      0.8292942756
Si              0.6404692282      0.8210069315      0.9981311751
Si              0.9667206614      0.9967580319      0.8661119927


--------------------------------------------------
 a-Si(1) - elastic constants
--------------------------------------------------

SOECs
       149.00  11
        50.29  12
        53.71  13
         3.03  15
        -2.32  16
        50.29  21
       148.52  22
        50.62  23
         2.88  26
        53.71  31
        50.62  32
```

142.98	33
1.28	34
1.20	36
1.28	43
47.20	44
3.03	51
48.00	55
1.40	56
-2.32	61
2.88	62
1.20	63
1.40	65
47.47	66

TOECs

-703.62	111
-314.84	112
-270.84	113
11.61	114
-23.58	115
-14.30	116
-314.84	121
-268.10	122
-197.62	123
-2.63	124
12.12	125
-26.83	126
-270.84	131
-197.62	132
-272.44	133
7.97	134
-16.93	135
1.19	136
11.61	141
-2.63	142
7.97	143
-19.32	144
-3.92	145
-5.68	146
-23.58	151
12.12	152
-16.93	153
-3.92	154
-104.76	155
-6.78	156
-14.30	161
-26.83	162
1.19	163
-5.68	164
-6.78	165
-114.42	166
-314.84	211
-268.10	212
-197.62	213
-2.63	214
12.12	215
-26.83	216
-268.10	221
-727.95	222
-250.63	223
-1.39	224

-24.32	225
-16.10	226
-197.62	231
-250.63	232
-248.99	233
9.85	234
-33.18	235
11.37	236
-2.63	241
-1.39	242
9.85	243
-93.11	244
-1.00	245
-3.54	246
12.12	251
-24.32	252
-33.18	253
-1.00	254
-25.26	255
5.15	256
-26.83	261
-16.10	262
11.37	263
-3.54	264
5.15	265
-118.31	266
-270.84	311
-197.62	312
-272.44	313
7.97	314
-16.93	315
1.19	316
-197.62	321
-250.63	322
-248.99	323
9.85	324
-33.18	325
11.37	326
-272.44	331
-248.99	332
-702.43	333
-12.89	334
12.12	335
-12.21	336
7.97	341
9.85	342
-12.89	343
-97.33	344
-9.76	345
-2.26	346
-16.93	351
-33.18	352
12.12	353
-9.76	354
-81.04	355
1.19	361
11.37	362
-12.21	363
-2.26	364
-37.70	366

11.61	411
-2.63	412
7.97	413
-19.32	414
-3.92	415
-5.68	416
-2.63	421
-1.39	422
9.85	423
-93.11	424
-1.00	425
-3.54	426
7.97	431
9.85	432
-12.89	433
-97.33	434
-9.76	435
-2.26	436
-19.32	441
-93.11	442
-97.33	443
-19.10	444
-3.88	445
-2.28	446
-3.92	451
-1.00	452
-9.76	453
-3.88	454
1.80	455
-30.72	456
-5.68	461
-3.54	462
-2.26	463
-2.28	464
-30.72	465
-4.33	466
-23.58	511
12.12	512
-16.93	513
-3.92	514
-104.76	515
-6.78	516
12.12	521
-24.32	522
-33.18	523
-1.00	524
-25.26	525
5.15	526
-16.93	531
-33.18	532
12.12	533
-9.76	534
-81.04	535
-3.92	541
-1.00	542
-9.76	543
-3.88	544
1.80	545
-30.72	546
-104.76	551

```
      -25.26  552
      -81.04  553
        1.80  554
       -7.55  555
        5.90  556
       -6.78  561
        5.15  562
      -30.72  564
        5.90  565
       -8.31  566
      -14.30  611
      -26.83  612
        1.19  613
       -5.68  614
       -6.78  615
     -114.42  616
      -26.83  621
      -16.10  622
       11.37  623
       -3.54  624
        5.15  625
     -118.31  626
        1.19  631
       11.37  632
      -12.21  633
       -2.26  634
      -37.70  636
       -5.68  641
       -3.54  642
       -2.26  643
       -2.28  644
      -30.72  645
       -4.33  646
       -6.78  651
        5.15  652
      -30.72  654
        5.90  655
       -8.31  656
     -114.42  661
     -118.31  662
      -37.70  663
       -4.33  664
       -8.31  665
      -18.39  666



--------------------------------------------------
 a-Si(2) - model structure
--------------------------------------------------

CELL_PARAMETERS (bohr)
  30.764941232  -0.106483652  -0.057602726
  -0.106721549  30.411092393  -0.254453393
  -0.057810168  -0.254765125  30.789643536
ATOMIC_POSITIONS (crystal)
Si               0.4595950615       0.3117226002       0.0321633428
Si               0.0657104069       0.0796086798       0.2116410254
Si               0.2536265479       0.0909348649       0.1243153936
Si               0.1029770228       0.4310772491       0.2083528348
```

Si	0.1158654864	0.1252461341	0.0872898943
Si	0.3297508297	0.3095433615	0.0998036243
Si	0.3422047591	0.4086097830	0.2047264574
Si	0.3757338015	0.0915071305	0.0423153134
Si	0.0364663723	0.2456100482	0.0550450775
Si	0.2990428335	0.2070002630	0.1976246594
Si	0.0127918638	0.3229204665	0.1798175298
Si	0.4960206606	0.1725285455	0.0418004103
Si	0.0428187365	0.0355624523	0.0013536763
Si	0.0949559853	0.0896783336	0.4351925772
Si	0.0912787611	0.1743888987	0.3192361113
Si	0.0484514448	0.4852997870	0.3320178445
Si	0.3678899067	0.2941030648	0.4870779173
Si	0.3222269508	0.0487255852	0.4293557952
Si	0.4424399444	0.2688714023	0.3686591089
Si	0.2356859267	0.2632136684	0.4536087000
Si	0.4631951301	0.3780617103	0.2820851419
Si	0.3684731115	0.0380129346	0.2941811090
Si	0.2132964199	0.2534234587	0.3096259702
Si	0.2216123300	0.3943501811	0.2796798781
Si	0.4193786626	0.1686855747	0.2631232521
Si	0.2201239310	0.1277670032	0.4943743679
Si	0.3875614658	0.0635214971	0.6416574962
Si	0.4236613976	0.1868910505	0.7089969346
Si	0.1703317600	0.3578614372	0.5390934003
Si	0.2135331266	0.1894019210	0.7322497851
Si	0.2448695018	0.0875388984	0.6301302144
Si	0.2030554037	0.3225977204	0.6757945179
Si	0.3951806416	0.4310316224	0.5347316174
Si	0.4216197837	0.2998426503	0.6191888115
Si	0.0278237618	0.3535366808	0.5299072439
Si	0.1957338157	0.4990179549	0.5178497070
Si	0.0178033689	0.4503878381	0.6397864432
Si	0.4393862037	0.0376398620	0.5110283073
Si	0.0719298288	0.3464985576	0.7238020132
Si	0.3244361910	0.3358244611	0.8642327092
Si	0.3465307791	0.0848660356	0.9028629911
Si	0.4568378950	0.3719056239	0.9039353633
Si	0.1074089030	0.0500409485	0.8751458330
Si	0.1031696233	0.1852883869	0.8266894621
Si	0.1151531728	0.2827944796	0.9407850958
Si	0.3295322799	0.1990509745	0.8154287460
Si	0.0549769742	0.3839969383	0.8606224383
Si	0.2512866039	0.4225995449	0.7732619070
Si	0.1536079011	0.4841012951	0.8622738077
Si	0.2444063851	0.3393692592	0.9850719315
Si	0.4532869042	0.0050013618	0.8455871907
Si	0.2068943555	0.4789430545	0.9937396203
Si	0.3117300725	0.8872931068	0.1169660749
Si	0.4868104413	0.5717221938	0.2021147174
Si	0.4748805486	0.7134417951	0.2045568745
Si	0.1360665746	0.6466998308	0.1076898661
Si	0.2615323951	0.6400184274	0.1835633063
Si	0.3474145185	0.7486625960	0.1460021403
Si	0.0380326014	0.7230302439	0.1824552739
Si	0.0348830831	0.8682032519	0.1707795110
Si	0.4270104350	0.9585673769	0.0714080353
Si	0.0801721370	0.9046087596	0.0421471759
Si	0.3574709570	0.5374366998	0.1474782045
Si	0.2598087807	0.9670236642	0.2219258804

Si	0.0994500978	0.5080957856	0.0893355793
Si	0.3470742295	0.7011218219	0.0090282993
Si	0.3339566000	0.5532478469	0.0076568723
Si	0.2214044587	0.8994152088	0.0064807843
Si	0.2495151116	0.6155265231	0.3239071047
Si	0.1966191143	0.7381521161	0.3719874053
Si	0.2822571224	0.8494217755	0.3225768100
Si	0.3726320129	0.6340507973	0.3962500958
Si	0.2459913852	0.9262191392	0.4359478208
Si	0.0628490418	0.7314116403	0.3239655982
Si	0.4613714672	0.8961056934	0.4923442859
Si	0.1091875138	0.9491754581	0.3991956259
Si	0.4211707610	0.8560630489	0.3613089106
Si	0.4685750548	0.7247818011	0.3445514119
Si	0.4885355673	0.9550065944	0.2829848493
Si	0.0120006844	0.8401716620	0.4074993652
Si	0.4529934350	0.5271446833	0.4405933171
Si	0.1192955850	0.9540679108	0.2555684253
Si	0.1858635348	0.4973854303	0.3757095264
Si	0.0325322739	0.7903744915	0.6404504837
Si	0.3163815723	0.5432390934	0.5824168465
Si	0.0592021023	0.9319022005	0.6553688296
Si	0.3982486638	0.9484284566	0.7282955402
Si	0.1983233984	0.9530889914	0.6533969781
Si	0.3881987116	0.8039420029	0.5742145157
Si	0.3334770314	0.6758022487	0.5253639759
Si	0.2575588415	0.8622838283	0.5600155320
Si	0.0809034957	0.5623974183	0.5753720983
Si	0.1303948225	0.6872087392	0.6295195196
Si	0.4531610394	0.8182471082	0.6981136049
Si	0.2996851762	0.5485330382	0.7231348794
Si	0.2034360828	0.7340328182	0.5173556335
Si	0.2066930415	0.6598690926	0.7463496379
Si	0.2737690597	0.7725239400	0.8069138726
Si	0.0249005960	0.9734351861	0.7880381087
Si	0.4223021699	0.5861699914	0.7889620192
Si	0.4102472977	0.7303670494	0.8008580403
Si	0.4396359280	0.5176516567	0.9168681491
Si	0.1262260543	0.6930603085	0.9686597514
Si	0.2652588243	0.9128992155	0.7727483402
Si	0.2415837491	0.7700633703	0.9450322621
Si	0.1106081355	0.6178976268	0.8450901690
Si	0.4521847063	0.7611009451	0.9325300913
Si	0.4587455938	0.9035711439	0.9462614730
Si	0.0259201942	0.7942550061	0.9672478908
Si	0.2373628081	0.9894105232	0.8932429711
Si	0.9097151750	0.0746278955	0.0435753879
Si	0.7004746899	0.3394731309	0.0334326760
Si	0.5742821257	0.3749697059	0.0921761484
Si	0.6548053771	0.0994196515	0.2174860113
Si	0.8925346636	0.3943494406	0.1555762453
Si	0.8123142919	0.2806172028	0.1006981146
Si	0.7610151231	0.2017212096	0.2115736177
Si	0.5368803677	0.1729380642	0.1796628136
Si	0.9248506586	0.0582424787	0.1885309011
Si	0.5683984629	0.3102745373	0.2139469085
Si	0.7776294561	0.4337085219	0.2330418321
Si	0.8984160525	0.2135166147	0.0082708516
Si	0.9791952039	0.2613282361	0.3046022284
Si	0.8614459619	0.1052793311	0.4119233057

Si	0.6987684075	0.4092168489	0.4544982970
Si	0.7692985882	0.1920274348	0.4779091872
Si	0.6867602344	0.2778715481	0.3993924434
Si	0.5610266971	0.1008915557	0.4611160046
Si	0.5584988950	0.2426235210	0.4420456679
Si	0.6000109419	0.0334550737	0.3370851166
Si	0.8763698053	0.1598921357	0.2815620051
Si	0.9697747307	0.3798240014	0.3990286601
Si	0.6993769186	0.3165250364	0.2663815458
Si	0.8440442279	0.4290714104	0.4636609694
Si	0.6269681389	0.4246551888	0.5742539425
Si	0.8392083759	0.2959616908	0.5537998114
Si	0.6728659472	0.1120944364	0.5516762085
Si	0.9849775382	0.2191610952	0.7326218805
Si	0.7167747488	0.4374878674	0.6827798227
Si	0.6514839991	0.2165696190	0.6503258022
Si	0.7683882130	0.3042496141	0.6774864978
Si	0.5590552656	0.2985400435	0.5757724276
Si	0.9666922353	0.2367048011	0.5835731782
Si	0.9341231458	0.0749338249	0.7401283758
Si	0.9524221920	0.0165197772	0.6057891213
Si	0.9741431684	0.1158883077	0.5038034953
Si	0.9189114769	0.4174738128	0.8415968389
Si	0.6774357593	0.2578927160	0.9203511173
Si	0.8690385534	0.2943202957	0.7815431473
Si	0.6196078459	0.4526361064	0.7839447384
Si	0.5839731989	0.0743545356	0.8270767806
Si	0.8144767819	0.2213767150	0.8865159012
Si	0.8193976677	0.0910843176	0.8265338394
Si	0.5760230881	0.3208416867	0.8341684109
Si	0.6091251750	0.1356850341	0.9567501434
Si	0.8178938228	0.0166035077	0.9494355702
Si	0.7109127868	0.0232485848	0.7624806605
Si	0.8794499929	0.4947916319	0.9563034602
Si	0.5569575622	0.1982230189	0.7538404471
Si	0.6803310547	0.0170301048	0.9944867188
Si	0.7481024728	0.4695770704	1.0002391202
Si	0.7071229336	0.7638215447	0.1866066060
Si	0.6706240950	0.9813369826	0.1318994773
Si	0.7765588567	0.8796472217	0.1374285822
Si	0.9251896761	0.6458895250	0.1228863381
Si	0.9075343024	0.7234000228	0.0061675150
Si	0.5747324751	0.7775866524	0.1301367672
Si	0.5402858984	0.9201827838	0.1509321175
Si	0.7909744694	0.6446634335	0.1753445552
Si	0.9005950256	0.9149636321	0.2093301788
Si	0.7228168590	0.5232619810	0.1329756030
Si	0.9531051125	0.5051484549	0.0779735566
Si	0.5803590287	0.5170720019	0.1114665590
Si	0.7950418163	0.8107609539	0.0094209129
Si	0.5668351418	0.7156386896	-0.0005163419
Si	0.6526199702	0.5282892672	0.3902889267
Si	0.6856816438	0.7912490781	0.3246165814
Si	0.6799318736	0.9261067341	0.3828553557
Si	0.8884571349	0.8743828504	0.3487528446
Si	0.5903091069	0.7340384039	0.4145675453
Si	0.8550184993	0.6325652037	0.3044612537
Si	0.8124072645	0.7504275910	0.3724993680
Si	0.8120707545	0.9698661523	0.4231999548
Si	0.5979408556	0.8620030565	0.4825138090

```
Si               0.9880328958       0.6130527754       0.3553127916
Si               0.7885636925       0.5152011081       0.3506240125
Si               0.5273344097       0.5029605276       0.3227809012
Si               0.7766111848       0.6809175519       0.4938809892
Si               0.9765472352       0.6230737654       0.4979680062
Si               0.6751538380       0.8577922198       0.6000394757
Si               0.8182681152       0.8000820839       0.5693418193
Si               0.8115792991       0.5446802096       0.6665759342
Si               0.5345507303       0.5468754568       0.7100460504
Si               0.5127486585       0.5077181959       0.5713023206
Si               0.9596502066       0.7670811929       0.5209509597
Si               0.5903922926       0.7894183502       0.6920272925
Si               0.8155605967       0.7822162413       0.7067287095
Si               0.6168900818       0.6511007500       0.6494050915
Si               0.8359615771       0.9447204386       0.5613616222
Si               0.7429553759       0.6588454311       0.7176267919
Si               0.8524834659       0.5619720899       0.5308570104
Si               0.9287463013       0.5086645134       0.7337586383
Si               0.7159672448       0.9965672183       0.6222975978
Si               0.9489211542       0.7582703814       0.7484755939
Si               0.6406802902       0.6321737207       0.4978993366
Si               0.5929699539       0.9294546546       0.9124427427
Si               0.6627972976       0.5277232296       0.9018879465
Si               0.6863528292       0.7496009038       0.9347963173
Si               0.5549107743       0.5725348963       0.9840845654
Si               0.6414262728       0.8278405289       0.8241191116
Si               0.7410809629       0.6395794783       0.8576318353
Si               0.8459270918       0.8866792402       0.9021512321
Si               0.8779893978       0.6269443639       0.9015594203
Si               0.9760592029       0.6283756148       0.7980485392
Si               0.9742918621       0.8550729024       0.8511786450
Si               0.7626302091       0.8897108666       0.7884649040

---------------------------------------------------
 a-Si(2) - elastic constants
---------------------------------------------------

SOECs
       142.05  11
        49.85  12
        48.14  13
        -1.80  14
         3.12  15
        49.85  21
       147.86  22
        50.80  23
         1.25  25
         2.07  26
        48.14  31
        50.80  32
       144.03  33
        -1.28  34
        -1.80  41
        -1.28  43
        46.23  44
        -1.83  45
        -1.58  46
         3.12  51
         1.25  52
        -1.83  54
```

43.43	55
1.58	56
2.07	62
-1.58	64
1.58	65
44.46	66

TOECs

-540.89	111
-249.15	112
-185.67	113
114.87	114
-13.39	115
13.87	116
-249.15	121
-242.47	122
-205.18	123
7.09	124
-36.17	125
-11.45	126
-185.67	131
-205.18	132
-172.97	133
38.76	134
-15.23	135
7.58	136
114.87	141
7.09	142
38.76	143
21.81	144
16.69	145
1.84	146
-13.39	151
-36.17	152
-15.23	153
16.69	154
-73.93	155
-6.31	156
13.87	161
-11.45	162
7.58	163
1.84	164
-6.31	165
-56.72	166
-249.15	211
-242.47	212
-205.18	213
7.09	214
-36.17	215
-11.45	216
-242.47	221
-767.21	222
-232.61	223
21.52	224
-1.54	226
-205.18	231
-232.61	232
-341.32	233
31.58	234
-19.96	235
-4.17	236

7.09	241
21.52	242
31.58	243
-133.29	244
-6.07	245
-9.78	246
-36.17	251
-19.96	253
-6.07	254
-25.96	255
-11.45	261
-1.54	262
-4.17	263
-9.78	264
-96.62	266
-185.67	311
-205.18	312
-172.97	313
38.76	314
-15.23	315
7.58	316
-205.18	321
-232.61	322
-341.32	323
31.58	324
-19.96	325
-4.17	326
-172.97	331
-341.32	332
-523.82	333
48.42	334
12.48	335
1.56	336
38.76	341
31.58	342
48.42	343
-78.90	344
10.71	345
21.82	346
-15.23	351
-19.96	352
12.48	353
10.71	354
-83.33	355
6.60	356
7.58	361
-4.17	362
1.56	363
21.82	364
6.60	365
-59.39	366
114.87	411
7.09	412
38.76	413
21.81	414
16.69	415
1.84	416
7.09	421
21.52	422
31.58	423

-133.29	424
-6.07	425
-9.78	426
38.76	431
31.58	432
48.42	433
-78.90	434
10.71	435
21.82	436
21.81	441
-133.29	442
-78.90	443
48.67	444
-7.31	445
14.77	446
16.69	451
-6.07	452
10.71	453
-7.31	454
15.73	455
-27.31	456
1.84	461
-9.78	462
21.82	463
14.77	464
-27.31	465
22.00	466
-13.39	511
-36.17	512
-15.23	513
16.69	514
-73.93	515
-6.31	516
-36.17	521
-19.96	523
-6.07	524
-25.96	525
-15.23	531
-19.96	532
12.48	533
10.71	534
-83.33	535
6.60	536
16.69	541
-6.07	542
10.71	543
-7.31	544
15.73	545
-27.31	546
-73.93	551
-25.96	552
-83.33	553
15.73	554
-10.03	555
15.61	556
-6.31	561
6.60	563
-27.31	564
15.61	565
-15.99	566

```
       13.87  611
      -11.45  612
        7.58  613
        1.84  614
       -6.31  615
      -56.72  616
      -11.45  621
       -1.54  622
       -4.17  623
       -9.78  624
      -96.62  626
        7.58  631
       -4.17  632
        1.56  633
       21.82  634
        6.60  635
      -59.39  636
        1.84  641
       -9.78  642
       21.82  643
       14.77  644
      -27.31  645
       22.00  646
       -6.31  651
        6.60  653
      -27.31  654
       15.61  655
      -15.99  656
      -56.72  661
      -96.62  662
      -59.39  663
       22.00  664
      -15.99  665
       -5.50  666



--------------------------------------------------
 a-Si(3) - model structure
--------------------------------------------------

CELL_PARAMETERS (bohr)
  30.596132081  -0.074891747  -0.310239051
  -0.074788284  30.863150338   0.259994358
  -0.310803343   0.260757625  30.160645114
ATOMIC_POSITIONS (crystal)
Si               0.0668089343       0.1122369907       0.0316357239
Si               0.4073209306       0.0880457676       0.0365568648
Si               0.7296292539       0.1391038597       0.1457130607
Si               0.7454220430       0.1338693855       0.0031244619
Si               0.1260777443       0.1800513292       0.1555841083
Si               0.1534781930       0.3134686276       0.1062548294
Si               0.5673281526       0.2854211758       0.0210315433
Si               0.9767769154       0.2361956340       0.0154058582
Si               0.9454795722       0.2650380205       0.1569665877
Si               0.0567704146       0.4060920086       0.0485326665
Si               0.3417470287       0.3963756137       0.0326010061
Si               0.6491981510       0.4973244587       0.0194328580
Si               0.7519439445       0.3645817555       0.1291281490
Si               0.6876702481       0.3606679284       0.0000385493
```

Si	0.8482232283	0.4707415108	0.1177782496
Si	0.9701820900	0.4083231019	0.1631727261
Si	0.1373407737	0.5252049102	0.0708806484
Si	0.0832557013	0.6471346386	0.0144017231
Si	0.2627634962	0.5145487784	0.0014509132
Si	0.3395043159	0.6297347899	0.0258579783
Si	0.6440555562	0.5714559356	0.1438455340
Si	0.9403658827	0.6607971420	0.0121540509
Si	0.1600979696	0.7687913517	0.0364662168
Si	0.5758340782	0.6975677155	0.1352394197
Si	0.7873868659	0.7981990581	0.1098788244
Si	0.8691167789	0.6863160114	0.1365679518
Si	0.0965386565	0.9778226178	0.0872771896
Si	0.3170059673	0.9760037854	0.0220671446
Si	0.2331641988	0.9869955314	0.1421505120
Si	0.3831107365	0.8513041388	0.0262719544
Si	0.6321158433	0.9292449998	0.1492778663
Si	0.8506412193	0.9276210752	0.1096044052
Si	0.2389997543	0.1098495544	0.2159519508
Si	0.4687852064	0.0826990195	0.1722902487
Si	0.3745780992	0.1562551040	0.2470579232
Si	0.5981571041	0.1496122720	0.2031296311
Si	0.8343821228	0.0324108857	0.3070192238
Si	0.9725218116	0.0347573301	0.2652577106
Si	0.0138962079	0.1713760392	0.2453415769
Si	0.4338257539	0.2883101873	0.2367819712
Si	0.5572790300	0.2791465444	0.1668899037
Si	0.8104358567	0.2493719536	0.1949572836
Si	0.0306733456	0.4881902511	0.2691354477
Si	0.2154802724	0.3880352221	0.2153118798
Si	0.3537859189	0.3910754284	0.1777340790
Si	0.6428358610	0.3791241358	0.2215453623
Si	0.5648792785	0.4977383111	0.2366737400
Si	0.1580902363	0.5225025200	0.2144925927
Si	0.2272789004	0.6492135069	0.2353950982
Si	0.3556302021	0.6264459024	0.1716677734
Si	0.4161242361	0.5069227065	0.2291969078
Si	0.7841001988	0.5765262581	0.1875321354
Si	0.8076450792	0.5654605277	0.3326313102
Si	0.9488853530	0.5893244138	0.3250261928
Si	0.0556487517	0.8162372320	0.2382182359
Si	0.1807123179	0.7761853868	0.1808165802
Si	0.4553619367	0.7175575242	0.2139065380
Si	0.6676348540	0.7935856899	0.1877447683
Si	0.6675642378	0.7563354856	0.3347724010
Si	0.9550878012	0.7140902940	0.2493621146
Si	0.2818901804	0.8752789688	0.2214742958
Si	0.4110999146	0.8450729153	0.1686015004
Si	0.5035430533	0.9485221476	0.2083287216
Si	0.7577992930	0.0052293471	0.1850305017
Si	0.9878696288	0.9305113979	0.1686911750
Si	0.0693518620	0.0021983145	0.3723072100
Si	0.0056008596	0.0741341224	0.4768277322
Si	0.1882007917	0.0826011312	0.3514218997
Si	0.2979192746	0.0279002996	0.4264414942
Si	0.3923412738	0.1294178061	0.3883353403
Si	0.5219846478	0.0671988081	0.4114158369
Si	0.6307489753	0.1403060799	0.3494712298
Si	0.7740764197	0.1347765559	0.3848157679
Si	0.0105193563	0.1945413977	0.3928349343

Si	0.1527014628	0.2082337987	0.4084899489
Si	0.1776480962	0.3346589371	0.3457581555
Si	0.3821909103	0.2608358244	0.4476696685
Si	0.4875260211	0.3194796480	0.3735459666
Si	0.6141077699	0.2656086127	0.4122765162
Si	0.8228658314	0.2681234987	0.3427911608
Si	0.9429443818	0.3153216537	0.4219737778
Si	0.0567653379	0.4034046444	0.3821174241
Si	0.1193345270	0.4823941171	0.4870840497
Si	0.2921407269	0.3794435336	0.4225213441
Si	0.4914464375	0.4517724364	0.4251485049
Si	0.3677788143	0.4950670275	0.3679504033
Si	0.7250802197	0.4733078723	0.4160992261
Si	0.7078503004	0.3501401501	0.3502893763
Si	0.1290574220	0.6231798074	0.4571115566
Si	0.3489115400	0.6249537268	0.4240119788
Si	0.5984283347	0.5301521905	0.3735983913
Si	0.9900810076	0.6471993791	0.4494879156
Si	0.2284869612	0.6846047548	0.3768786637
Si	0.2169550513	0.8202509488	0.4277587242
Si	0.4484823526	0.7101150080	0.3607980547
Si	0.5773026591	0.6665719791	0.3994876329
Si	0.7849860698	0.6922458388	0.3895997947
Si	0.8761920797	0.7892555390	0.3411964876
Si	0.9852338203	0.7953064481	0.4508639463
Si	0.0876195525	0.8606796695	0.3755634048
Si	0.3136403542	0.9008003601	0.3633457060
Si	0.4384013436	0.8446942866	0.4081001658
Si	0.6624349787	0.8843839131	0.3918506216
Si	0.5332274478	0.9378941570	0.3522121855
Si	0.8008608851	0.9079698730	0.3618641048
Si	0.8705783652	0.8784631149	0.4859796565
Si	0.0640553354	0.1274302863	0.5977397946
Si	0.3026910028	0.0261183126	0.5740521170
Si	0.2899373397	0.1619871664	0.6143223057
Si	0.5374400960	0.0547724602	0.5554475115
Si	0.6743478194	0.0160923432	0.5815351897
Si	0.7855786142	0.0889791575	0.5241126607
Si	0.8992689137	0.0032542403	0.5425789538
Si	0.1704399163	0.2156063427	0.5549133062
Si	0.4032836637	0.2441918596	0.5905791091
Si	0.5296348364	0.1795040905	0.6183287255
Si	0.6288540158	0.2602466322	0.5584254509
Si	0.8341888438	0.1668017458	0.6354053997
Si	0.9671119449	0.2197981039	0.6467956633
Si	0.0471647897	0.4256209518	0.5984562330
Si	0.1597695772	0.3402423435	0.6253066992
Si	0.2516866169	0.4260209420	0.5567248718
Si	0.3774737747	0.3792076633	0.6226639828
Si	0.4854389178	0.4537769294	0.5683655400
Si	0.5983290249	0.3840342225	0.6165233797
Si	0.7162108294	0.4592749430	0.5667435051
Si	0.8142393240	0.3792412724	0.6409932371
Si	0.9354738704	0.3399698403	0.5706408430
Si	0.2668032500	0.5471310913	0.6389482352
Si	0.3581300692	0.6335245175	0.5696602141
Si	0.4903255566	0.5897422801	0.6059075749
Si	0.7034457435	0.5963407219	0.5918823224
Si	0.9354606512	0.6353276775	0.5933710573
Si	0.1348121759	0.7110521617	0.5698039252

Si	0.0051526897	0.7593983893	0.5934938340
Si	0.2204975082	0.8233904750	0.5765779668
Si	0.3465949040	0.7655849802	0.6174541680
Si	0.5900869989	0.6701533530	0.5439341676
Si	0.5731932726	0.8017668852	0.5960961925
Si	0.8034150221	0.6752968372	0.5338464550
Si	0.7896252219	0.8078613097	0.5796793758
Si	0.2098798642	0.9483190717	0.6547511846
Si	0.4443920515	0.8462544925	0.5541763381
Si	0.4318024674	0.9738453602	0.6130668031
Si	0.6737969044	0.8812408262	0.5365724842
Si	0.1050045189	0.0337231096	0.7053595429
Si	0.1331669290	0.1405056973	0.7984922917
Si	0.4710227981	0.0766644199	0.8233527426
Si	0.5818209188	0.1318293984	0.7482504557
Si	0.6872657114	0.0364060613	0.7254197211
Si	0.8246374001	0.0769955174	0.7457881387
Si	0.0127453551	0.2265793385	0.7906809943
Si	0.2606502305	0.1854385714	0.7518281204
Si	0.2212685715	0.3210489380	0.7569813896
Si	0.4409870980	0.3117934544	0.8289293234
Si	0.6598705706	0.2435697485	0.8001959687
Si	0.7337871889	0.2603716302	0.6608803620
Si	0.3456341468	0.3922191558	0.7608689385
Si	0.5649254090	0.3442569511	0.7592855791
Si	0.5844527888	0.4732493637	0.8120583693
Si	0.8211729234	0.4052689112	0.7887488750
Si	0.0293021991	0.5429451255	0.6717215650
Si	0.1511346646	0.6203564883	0.6758524489
Si	0.3094750380	0.5254661505	0.7789071499
Si	0.4125551754	0.6204468721	0.8110657576
Si	0.5330981656	0.5900915115	0.7458830144
Si	0.7406994155	0.6173942942	0.7276752647
Si	0.7231913242	0.5032882324	0.8092516319
Si	0.9823370134	0.5541870600	0.8069881739
Si	0.8817360709	0.6357359637	0.7389876194
Si	0.0348588869	0.8025988426	0.7290245985
Si	0.1430172458	0.7163781792	0.7837514091
Si	0.3777131917	0.7485555858	0.7618127599
Si	0.6351447253	0.6883075885	0.7858465052
Si	0.5948627132	0.8149498043	0.7396165404
Si	0.9075269502	0.7647288023	0.7835729185
Si	0.0152993299	0.9371332729	0.7633565093
Si	0.2735928584	0.9262696350	0.7888111400
Si	0.4763780627	0.8428021440	0.8094979783
Si	0.4139827447	0.9615424321	0.7596885747
Si	0.6987408055	0.9043977211	0.7785321467
Si	0.8177594800	0.8545575802	0.7140593472
Si	0.8969583524	0.9708441165	0.6877778584
Si	0.1758210691	0.1086610761	0.9350966919
Si	0.5256185773	0.0634029478	0.9582224732
Si	0.8171702821	0.0080110787	0.9874501614
Si	0.9116051591	0.1368628673	0.8450550191
Si	0.9517354497	0.0397813568	0.9466669243
Si	0.3041570740	0.1752986280	0.9684848479
Si	0.2433239178	0.3029837461	0.9940326847
Si	0.3753726811	0.1826183465	0.8407638439
Si	0.6157876457	0.1745467513	0.9447118780
Si	0.7621971024	0.3115512920	0.8824629753
Si	0.8486582248	0.2148431868	0.9459296267

```
Si              0.0241443581       0.3374262136       0.9129474824
Si              0.1640145892       0.3645248873       0.8812218405
Si              0.4626092907       0.3673614580       0.9614517297
Si              0.5283404483       0.4918683414       0.9439044732
Si              0.9294707152       0.4436853332       0.8787496881
Si              0.0970982045       0.6133066541       0.8745980376
Si              0.1990526442       0.5078145694       0.8706924520
Si              0.4574833740       0.6140880083       0.9499928543
Si              0.7431071623       0.5638943152       0.9377342932
Si              0.8759795273       0.5306252152       0.9848386284
Si              0.2624973932       0.7861247118       0.8318026232
Si              0.2878091214       0.7553273345       0.9761428715
Si              0.5543568688       0.7118533812       0.9914228715
Si              0.6792833300       0.6904577843       0.9249230496
Si              0.7664511249       0.7956591726       0.9658852018
Si              0.9052960517       0.7816561242       0.9275939856
Si              0.1160438367       0.8921987907       0.9746419978
Si              0.2184583508       0.9749830189       0.9134611092
Si              0.5057512895       0.8412001221       0.9539920459
Si              0.5993187622       0.9430430073       0.9994950886
Si              0.7195083569       0.9210655245       0.9220362679
Si              0.9879743851       0.9073034562       0.9074569382

---------------------------------------------------
 a-Si(3) - elastic constants
---------------------------------------------------

SOECs
       154.65  11
        51.55  12
        46.22  13
        -1.45  15
        51.55  21
       152.33  22
        42.38  23
         1.05  25
        46.22  31
        42.38  32
       157.14  33
         1.43  35
        39.97  44
        -1.13  46
        -1.45  51
         1.05  52
         1.43  53
        44.44  55
        -3.12  56
        -1.13  64
        -3.12  65
        53.55  66
TOECs
      -832.97  111
      -255.81  112
      -205.16  113
       -16.45  114
        -2.09  115
       -41.09  116
      -255.81  121
      -280.89  122
      -195.15  123
```

-17.04	124
-2.85	125
13.36	126
-205.16	131
-195.15	132
-239.46	133
-17.08	134
-16.67	135
3.64	136
-16.45	141
-17.04	142
-17.08	143
-66.29	144
-5.36	145
4.76	146
-2.09	151
-2.85	152
-16.67	153
-5.36	154
-72.07	155
28.99	156
-41.09	161
13.36	162
3.64	163
4.76	164
28.99	165
-145.66	166
-255.81	211
-280.89	212
-195.15	213
-17.04	214
-2.85	215
13.36	216
-280.89	221
-849.34	222
-241.63	223
-16.99	224
58.55	225
-85.27	226
-195.15	231
-241.63	232
-264.62	233
-14.01	234
-3.67	235
3.99	236
-17.04	241
-16.99	242
-14.01	243
-125.67	244
-14.34	245
-2.85	251
58.55	252
-3.67	253
-14.34	254
-43.60	255
5.12	256
13.36	261
-85.27	262
3.99	263
5.12	265

-171.09	266
-205.16	311
-195.15	312
-239.46	313
-17.08	314
-16.67	315
3.64	316
-195.15	321
-241.63	322
-264.62	323
-14.01	324
-3.67	325
3.99	326
-239.46	331
-264.62	332
-709.48	333
-72.22	334
-1.77	335
17.62	336
-17.08	341
-14.01	342
-72.22	343
-94.14	344
-1.95	346
-16.67	351
-3.67	352
-1.77	353
-125.08	355
-3.62	356
3.64	361
3.99	362
17.62	363
-1.95	364
-3.62	365
-19.54	366
-16.45	411
-17.04	412
-17.08	413
-66.29	414
-5.36	415
4.76	416
-17.04	421
-16.99	422
-14.01	423
-125.67	424
-14.34	425
-17.08	431
-14.01	432
-72.22	433
-94.14	434
-1.95	436
-66.29	441
-125.67	442
-94.14	443
5.65	444
18.26	445
10.75	446
-5.36	451
-14.34	452
18.26	454

22.45	455
-18.16	456
4.76	461
-1.95	463
10.75	464
-18.16	465
-2.09	511
-2.85	512
-16.67	513
-5.36	514
-72.07	515
28.99	516
-2.85	521
58.55	522
-3.67	523
-14.34	524
-43.60	525
5.12	526
-16.67	531
-3.67	532
-1.77	533
-125.08	535
-3.62	536
-5.36	541
-14.34	542
18.26	544
22.45	545
-18.16	546
-72.07	551
-43.60	552
-125.08	553
22.45	554
-1.26	555
-34.39	556
28.99	561
5.12	562
-3.62	563
-18.16	564
-34.39	565
7.18	566
-41.09	611
13.36	612
3.64	613
4.76	614
28.99	615
-145.66	616
13.36	621
-85.27	622
3.99	623
5.12	625
-171.09	626
3.64	631
3.99	632
17.62	633
-1.95	634
-3.62	635
-19.54	636
4.76	641
-1.95	643
10.75	644

```
      -18.16  645
       28.99  651
        5.12  652
       -3.62  653
      -18.16  654
      -34.39  655
        7.18  656
     -145.66  661
     -171.09  662
      -19.54  663
        7.18  665
       31.49  666


--------------------------------------------------
 a-SiO2(1) - model structure
--------------------------------------------------

CELL_PARAMETERS (bohr)
  19.763891173  -0.442864609  -0.259019394
  -0.435573406  18.651265875  -0.183400405
  -0.258262916  -0.181548879  18.644171985
ATOMIC_POSITIONS (angstrom)
O             5.0340093441        8.2911955421        4.5020203955
O             3.0352931534        6.6794658641        5.0660955744
O             0.8317674317        3.4435760703        8.5452336169
O             4.5316843239        6.8060857007        0.0520825212
O             8.9479076784        7.0015464443        8.3108027734
O             1.8945133912        3.9822005748        1.1080149981
O             9.5923875113        2.6631440188        3.6243202726
O            10.0254502448        7.6870537826        4.8213065349
O             0.3939389434        5.7710211608       -0.0849571227
O             6.4041246804        6.3795070504        8.1419958546
O             7.1514529218        3.1723340681        9.2531541053
O             7.8557168251        6.7027352047        5.9087098353
O             1.6399793532        4.9982248468        6.5404506198
O             5.9967826287        0.2903758219        2.8041155819
O             1.8684703670        7.5704436493        7.3044773012
O             3.8961074817        5.8863707714        7.4556681416
O             5.4700145445        5.6372051164        4.8806969064
O             0.1534048983        9.4969006774        7.0306385044
O             4.4327763224        3.2669386632        4.6102682917
O             8.3170610378        4.6368002455        7.4567842340
O             4.5822522832        4.4470198962        1.1645454507
O             6.6269394348        6.1858014729        1.4369700102
O             8.6197541612       -0.2375484240        5.7116290102
O             1.8833254565        2.6208363346        4.0956372826
O             0.3370429494        0.6262537579        4.6129485371
O             7.0371888198       -0.1700520046        7.8134020401
O             4.2755374346        6.5640718034        2.6910057621
O             2.4274474984        0.2005796283        8.5541461710
O             7.9170596520        3.9047735553        2.0186859403
O             5.9380690152        3.7544668903        6.7931833061
O             9.1443366409        5.2378380924        4.0439379099
O             0.5461545448        8.3028499927        9.4718294887
O             7.8609807591        7.4287458474        3.3613999027
O             5.6666535497        0.2486731538        0.1697234060
O             4.7266943130        1.0812872793        7.7232151642
O             7.9675779828        2.0691529806        6.9336453188
O             7.8147286223        1.4387481052        1.1937172718
```

O	7.3309761198	8.7265820402	1.1329921979
O	9.7739993479	3.1254857713	0.4502288795
O	3.4221836576	2.4625049819	9.5191339576
O	7.1213826983	3.5884548387	4.4661577367
O	3.8604494818	0.7026244579	4.3015497915
O	6.2591730988	0.7296346727	5.3843500596
O	3.6427654663	2.3048737160	2.2527585705
O	2.6416490608	2.4727993452	6.9989947636
O	0.2037591149	2.8576110754	6.0917659996
O	9.2179025263	6.9910909195	1.1017422584
O	4.6121864396	8.3465952531	7.9453164704
Si	1.2086049913	8.8436259272	8.0724388022
Si	7.3063657880	3.3741645013	7.6276926232
Si	2.5850320757	6.3104556985	6.5858130217
Si	8.1855674588	2.9449854718	0.7285107413
Si	1.3530832634	3.4832413204	7.0350336188
Si	6.7490170687	0.2372393994	1.3792983333
Si	0.6232159672	4.1833594581	0.1072087657
Si	7.4733316330	0.6031005471	6.4469218822
Si	3.4419544779	2.2180042530	3.8569887617
Si	5.7285140063	4.0481989427	5.1875895195
Si	9.9393731793	6.8560152514	9.5874204538
Si	8.4503663709	3.8374415478	3.5588603362
Si	7.7621813240	7.3089938442	1.7482404121
Si	4.4636551662	6.7832658578	4.2904144139
Si	0.3809930268	2.2539223178	4.5978086885
Si	3.3980395782	3.3414718344	1.0426055133
Si	9.9329770524	9.1994742487	5.4323555652
Si	4.8595230972	6.8073745386	8.3711450616
Si	8.7310511376	6.7327112059	4.5397380464
Si	5.5419024691	-0.1223291224	8.4399164450
Si	3.2527545418	1.5615394675	8.2008848699
Si	5.3421743790	0.0058639686	4.2624619352
Si	5.0136061566	5.9904317358	1.3639781788
Si	7.8559191312	6.1990429881	7.4428268263

----
a-SiO2(1) - elastic constants

SOECs

72.36	11
20.36	12
19.56	13
-4.29	14
-4.30	15
2.05	16
108.42	22
15.45	23
-1.69	24
-3.78	25
12.36	26
118.33	33
-1.24	34
0.09	35
4.61	36
33.45	44
2.86	45
4.45	46
30.79	55

-4.23	56
42.75	66

TOECs

137.78	111
294.73	112
36.58	113
-5.45	114
-33.05	115
-1.04	116
324.96	122
95.61	123
55.42	124
2.99	125
2.82	126
6.98	133
17.67	134
-27.08	135
28.80	136
-12.59	144
2.99	145
-14.46	146
58.49	155
11.10	156
-6.34	166
166.79	222
242.62	223
29.54	224
96.13	225
13.49	226
94.50	233
19.62	235
39.17	236
-15.00	244
12.67	245
-47.08	246
127.81	255
66.34	256
-28.79	266
-442.15	333
17.90	334
-30.20	335
38.66	336
22.99	344
-34.02	345
-8.23	346
70.80	355
-2.74	356
69.21	366
-26.13	444
-1.25	445
36.39	446
-4.28	455
-35.80	456
7.58	466
-37.76	555
39.03	556
-4.66	566
70.53	666

```
-------------------------------------------------------
 a-SiO2(2) - model structure
-------------------------------------------------------

CELL_PARAMETERS (bohr)
  24.749055130  -0.023280894  -0.056199256
  -0.022523098  24.146543598  -0.541213891
  -0.055198958  -0.533823887  24.488938278
ATOMIC_POSITIONS (angstrom)
O            12.2722274641        4.1797687072        0.2847508248
O             5.5133360575        3.2092519051        3.4243854194
O             7.6987369927        0.5692568359        0.0042161624
O             3.4134801462       12.3152389299       10.5428214014
O             8.0039162207        3.7149016214        2.8212552148
O             1.0540783045       11.6807290608        9.7448492470
O             3.6010300162       11.9510353118        3.1425139287
O             4.5254462422        1.9459412214        8.7009286495
O            10.0891870865        9.0689732234       11.1742492117
O            10.6679981878        2.1255384379        8.9968303759
O             4.9261300559        6.6603215796        6.3008199022
O             6.3633596778        1.4977266250        6.9175468264
O            10.5723000425        8.6875717760        8.5999264199
O             3.1923296652        8.5628792041        7.0982456406
O             2.6909814815        5.1513414138        2.3034982240
O             9.9416170365        6.4592948536        7.2602201361
O             6.9684048524        2.5799463539        9.2587148024
O            12.5396256675       11.9288658958        1.5005649698
O            12.0429412548        0.8571799228        3.8830060187
O             3.8929672859        3.3622047017       11.7443492542
O             6.8474160680       10.7493021681        4.1746593793
O             3.7009735739        1.6084403289        2.4313381785
O             0.2029504575        9.3627017920        1.9126878338
O             2.2442353581        7.7206061026        2.3891477028
O             0.5448110548        2.0865574785        5.6455627787
O             2.0133721418        9.9916754186        3.6883354954
O             7.3095937981       10.1871307245        9.0475678984
O             1.1372531856       10.1450698774        6.6292383915
O             9.5028302002        7.4715626351        1.5809573757
O             9.1037458660        5.0268145798        0.8898030485
O             6.3022284385       -0.0460047897        8.9562788520
O            12.3656864912        1.7291634415        1.4393700308
O            11.2429521605       10.4494206101        3.1575526138
O             2.3599916323        3.5863362804        4.4267280542
O             7.0381591535        8.9665663321        2.3111940081
O             0.3945811211        3.7364270205        2.6023837891
O             4.3481086780        6.6423450617        3.6953047145
O             9.8873263676        1.2384534636       11.5413362021
O            12.8305778693        5.1324071711       10.8767009845
O             5.4902961736        4.8048806898        9.5784496591
O            11.9725385888        6.1583623243        5.6838637092
O             3.5554963615       10.7604037028        5.6167403099
O             8.9342667813        6.5633252270       11.9441496434
O             9.3794268402       10.7973600012        4.9336138989
O             1.4182620833        3.6462858616       11.9245862731
O            11.7068168906        9.8873705423        5.6896478201
O             0.3267316260        8.1412762231        4.3047118563
O            10.4649469721        5.6116540678       10.0417492589
O            13.0351439507       12.2260412101        5.5110519658
O            10.9135378065        3.9817530788        7.0221862481
O            11.1031344314        9.9030530982        0.5785880367
```

O	8.3416671132	12.2464031306	10.2309195679
O	7.5130153113	2.9506615290	5.1594076709
O	12.2106394185	7.6068465115	10.3329988824
O	5.8731105952	11.3874225495	10.8246742465
O	4.4645035520	7.1143139877	8.9208935357
O	10.0353098610	8.3353244316	4.1329847941
O	12.2379483477	11.3567532172	7.8206149933
O	3.0535842380	5.4763190857	10.5048236070
O	5.3321633589	2.8759188057	0.8106035309
O	2.8951732010	1.0968088816	4.9438192226
O	5.2379314860	6.6201408633	11.4844836089
O	0.9050290125	5.6829033644	4.2024519606
O	6.5181469190	3.5593029656	11.6944792326
O	2.2804310239	0.6793373167	8.6068141061
O	12.3488625578	5.8358516272	8.2901948766
O	8.9508189707	2.0989269511	7.0803262445
O	11.7350710255	6.6657565376	3.0335036632
O	10.7572622378	3.6356857089	2.3989665263
O	1.3564597248	0.6076807019	2.8234013047
O	11.3309761425	1.3974262124	6.4682183637
O	6.5134341295	5.3476869688	4.5478895146
O	6.7750064829	11.2635891697	1.1469763602
O	5.8494766378	8.9454213490	0.0273571672
O	8.4587384618	9.5923410343	0.1280456041
O	7.3694278309	10.9762192188	6.6470344176
O	12.1869776745	2.5288790372	11.0474023417
O	9.4119687379	5.7260784349	3.4837256237
O	2.3401879586	3.1516973359	9.5315856147
O	4.6431186397	9.5871990100	3.4495989962
O	6.9892689472	6.6609301374	0.6389440869
O	8.5019039475	7.2379135914	9.3906357904
O	8.0220894857	0.2565589671	5.3374194939
O	12.1349005507	10.2291328132	10.1764141652
O	2.6968547363	2.7814240467	6.9700319616
O	5.3495318265	1.2647360123	11.3436507921
O	4.3749698318	6.8679693051	1.0389072468
O	11.7994434911	0.0662936256	10.2120804424
O	8.3508227079	8.5796375337	7.1532545498
O	3.1254608083	10.9662019112	8.2283550387
O	5.7366869497	8.9281532201	7.3947113049
O	9.7578534539	11.5182224958	12.3219318133
O	8.1085662257	4.7576235885	10.0873258768
O	6.4136150222	8.0302110120	4.6239213662
O	6.3100173291	0.9398040386	2.2564758034
O	4.9594996684	12.6937859199	-0.0208290030
Si	12.1975606005	3.3380048075	1.6735760846
Si	11.2550062108	8.8989633739	10.0662801069
Si	3.4380467237	6.6119348162	2.3490761599
Si	8.9282855013	-0.0331670602	11.9464869226
Si	8.9691603578	6.0634532861	10.4132391677
Si	5.2615463073	2.6660447924	12.1738977425
Si	9.3565615438	7.7273372377	8.0862315074
Si	7.0285606894	9.7035733187	0.8638528337
Si	4.5854538098	7.7848071028	7.4430901872
Si	11.2997536564	5.5753094775	7.0523094937
Si	5.6008897859	7.3227195866	0.0603627656
Si	4.5522464623	6.0285393345	10.1277952754
Si	2.5026303195	12.1084188847	9.2050649342
Si	6.9669031707	11.6394993142	9.6540641316
Si	2.6525335725	3.9387088157	10.9138329415

Si	12.9067994149	3.7817375233	11.7655507918
Si	12.5792160701	11.5564519876	9.3868973716
Si	2.1239907269	2.4063375337	5.5090360257
Si	5.2091108483	2.1510018712	2.2437170772
Si	5.5511660408	6.6425878336	4.7865378793
Si	2.9579020927	2.1455579633	8.4339555334
Si	2.7435976572	10.1164496303	6.8856969110
Si	12.8575761014	0.5773110184	2.4870861435
Si	12.7400784011	6.6450188157	4.3363176771
Si	6.2386762041	9.3380900379	3.6676823668
Si	11.0965968932	1.5006801902	10.4417799174
Si	3.4797894594	10.5821679758	3.9953991453
Si	6.7504829996	3.9265589391	10.1281029794
Si	6.8844989585	3.8573776401	3.9987363530
Si	1.1935842738	8.7613151286	3.0574927517
Si	12.0026092771	6.0627659290	9.8714090815
Si	6.4684386306	12.7596429342	0.6679971982
Si	7.1759095947	9.6356734998	7.5325163698
Si	12.4991272671	0.9389500839	5.4494950742
Si	10.1540155110	7.1157353099	3.0507897953
Si	12.0654755057	10.4050767540	1.7709246339
Si	10.4955271662	2.4373607139	7.4064620609
Si	8.6179267292	6.5377374340	0.5306252617
Si	10.5951211058	9.8384842698	4.4961771942
Si	6.0468557748	1.4850777987	8.4946570302
Si	4.8171832730	12.5213268551	11.3159576023
Si	1.5999263048	4.5174089750	3.3414463815
Si	12.8054037707	10.8739760254	6.3842795903
Si	9.3610902555	4.4806762210	2.4044058507
Si	9.8364963186	9.9034783554	12.5375161831
Si	2.8717696699	0.6096229091	3.3896162308
Si	7.7419045421	1.6753546368	6.0986162002
Si	7.9148680492	11.4194499480	5.1889940226

----

a-SiO2(2) - elastic constants

SOECs

78.36	11
31.49	12
23.08	13
-4.16	14
-5.05	15
2.87	16
90.52	22
12.41	23
-5.21	24
0.78	25
-1.04	26
60.65	33
-5.15	34
-1.27	35
1.17	36
30.90	44
0.94	45
0.61	46
29.71	55
-2.05	56
29.59	66

TOECs

200.19	111
-51.37	112
-140.08	113
26.88	114
33.56	115
228.57	116
52.16	122
-194.03	123
31.87	124
-26.04	125
66.35	126
88.83	133
13.59	134
-6.27	135
-56.45	136
6.49	144
-7.82	145
-2.40	146
-6.41	155
-12.09	156
161.41	166
383.80	222
68.04	223
-11.64	224
25.52	225
-24.78	226
408.91	233
77.94	234
13.81	235
-39.55	236
13.83	244
-14.80	245
7.57	246
7.17	255
2.98	256
8.96	266
-62.65	333
-65.02	334
14.69	335
60.64	336
-14.81	345
10.42	346
-13.12	355
-4.23	356
-39.76	366
10.03	444
9.80	445
-10.25	446
-15.69	455
-4.42	456
-25.67	466
9.33	555
-1.94	556
10.22	566
99.72	666
